# Interferon- and STING-independent induction of type I interferon stimulated genes during fractionated irradiation

**DOI:** 10.1186/s13046-021-01962-2

**Published:** 2021-05-08

**Authors:** Ruben S. A. Goedegebuure, Esther A. Kleibeuker, Francesca M. Buffa, Kitty C. M. Castricum, Syed Haider, Iris A. Schulkens, Luuk ten Kroode, Jaap van den Berg, Maarten A. J. M. Jacobs, Anne-Marie van Berkel, Nicole C. T. van Grieken, Sarah Derks, Ben J. Slotman, Henk M. W. Verheul, Adrian L. Harris, Victor L. Thijssen

**Affiliations:** 1 Department of Medical Oncology, Cancer Center Amsterdam, Amsterdam UMC, location VUmc, Amsterdam, The Netherlands; 2 Oncode Institute, Utrecht, The Netherlands; 3 Department of Molecular Oncology, University of Oxford, Oxford, UK; 4 Department of Radiation Oncology, Cancer Center Amsterdam, Amsterdam UMC, location VUmc, Amsterdam, The Netherlands; 5 Department of Gastroenterology, Cancer Center Amsterdam, Amsterdam UMC, location VUmc, Amsterdam, The Netherlands; 6 Department of Gastroenterology, Noord West Ziekenhuisgroep, Alkmaar, The Netherlands; 7 Department of Pathology, Cancer Center Amsterdam, Amsterdam UMC, location VUmc, Amsterdam, The Netherlands; 8 Department of Medical Oncology, Radboud UMC, Nijmegen, The Netherlands

**Keywords:** Radiotherapy, Type I interferons, Immune response

## Abstract

**Background:**

Improvement of radiotherapy efficacy requires better insight in the dynamic responses that occur during irradiation. Here, we aimed to identify the molecular responses that are triggered during clinically applied fractionated irradiation.

**Methods:**

Gene expression analysis was performed by RNAseq or microarray analysis of cancer cells or xenograft tumors, respectively, subjected to 3–5 weeks of 5 × 2 Gy/week. Validation of altered gene expression was performed by qPCR and/or ELISA in multiple cancer cell lines as well as in pre- and on-treatment biopsies from esophageal cancer patients (NCT02072720). Targeted protein inhibition and CRISPR/Cas-induced gene knockout was used to analyze the role of type I interferons and cGAS/STING signaling pathway in the molecular and cellular response to fractionated irradiation.

**Results:**

Gene expression analysis identified type I interferon signaling as the most significantly enriched biological process induced during fractionated irradiation. The commonality of this response was confirmed in all irradiated cell lines, the xenograft tumors and in biopsies from esophageal cancer patients. Time-course analyses demonstrated a peak in interferon-stimulated gene (ISG) expression within 2–3 weeks of treatment. The response was accompanied by a variable induction of predominantly interferon-beta and/or -lambda, but blocking these interferons did not affect ISG expression induction. The same was true for targeted inhibition of the upstream regulatory STING protein while knockout of STING expression only delayed the ISG expression induction.

**Conclusions:**

Collectively, the presented data show that clinically applied fractionated low-dose irradiation can induce a delayed type I interferon response that occurs independently of interferon expression or STING signaling. These findings have implications for current efforts that aim to target the type I interferon response for cancer treatment.

**Supplementary Information:**

The online version contains supplementary material available at 10.1186/s13046-021-01962-2.

## Background

Radiotherapy (RTx) remains a key modality of cancer treatment. For over a century, the clinical benefit of RTx has increased due to technical innovations that allow a more precise and targeted delivery of ionizing radiation to malignant tissues [1]. In addition, better insight in the biological and cellular response mechanisms to RTx has instigated the development of combination treatments that further improved the therapeutic outcome [2–4]. Many of the combination therapies comprise drugs that target tumor cell response mechanisms involved in radiotolerance or radioresistance [5, 6]. The efficacy of such combination therapies depends on adequate dose-scheduling and timing of the different treatment modalities [6]. To further improve combination radiotherapy, it is vital to better understand cellular and molecular responses and their time course during treatment. Gaining insight in the dynamic responses to radiotherapy is especially relevant for patients that are treated with a daily dose of irradiation for several weeks (conventional fractionated radiotherapy). Indeed, exploring molecular responses to irradiation has been recognized as an unmet need to develop rational approaches of combination radiotherapies [6].

While radiation-induced changes of gene expression have been explored previously [7–10], most studies have been aimed at identifying mechanisms that are involved in the development of acquired radioresistance. The induction of such a radioresistant phenotype usually requires irradiation schedules that are not commonly used in a clinical setting. Consequently, there is still only limited insight in the dynamics of cellular and molecular responses that actually occur during the time course of clinically applied low-dose fractionated irradiation. This lack of knowledge hampers the development and optimization of effective combination treatments with radiotherapy. Recently, we have shown that conventional fractionated RTx (daily 2 Gy irradiation, 5 days per week, up to 6 weeks) can induce a reversible radiotolerant phenotype in cancer cells in vitro; a response we coined as adaptive radioresistance. This response occurs in cancer cells of different origin and is characterized by convergence of clonogenic survival to a steady state level during treatment [11]. The observation that the surviving cells display the same radiosensitivity as non-irradiated cells following treatment suggests that cancer cells do not acquire radioresistance as a genetic trait. Possibly, a balance between cell death and repopulation occurs with cells adopting a phenotype that allows them to tolerate repetitive cycles of irradiation. This might represent a radioresistance mechanism with potentially clinical implications which urged us to further study the molecular pathways that are triggered during conventional low-dose fractionated irradiation.

Here, we report that clinically applied fractionated irradiation is accompanied by the induction of a type I interferon response which is characterized by the increased expression of interferon stimulated genes in vitro, in vivo and in esophageal cancer patients. Importantly, the observed response occurs independently of induction of specific type I/III interferon expression or upstream activation of the STING signaling pathway. Our findings have implications for current efforts to develop drugs that target the type I interferon response and warrant further investigation into the role of the type I interferons and interferon stimulated genes during fractionated radiotherapy.

## Methods

### Cell culture

The high-grade astrocytoma cell line D384 (grade III), colorectal cancer cell lines HT29, RKO, SW480, COLO320 and HCT116 and esophageal cancer cell line OE19 were cultured in Dulbecco’s Modified Eagle Medium (DMEM), supplemented with 10% fetal calf serum, 100 IU/mL penicillin and 100 μg/mL streptomycin. Cells were maintained at 37 °C and 5%CO2 under humidified conditions. Cell lines were authenticated by STR profiling (BaseClear, Leiden, The Netherlands) and were repeatedly found negative for mycoplasm infection as checked by PCR.

### In vitro and in vivo irradiation

Irradiation of cultured cells in vitro was performed with γ-radiation using a ^60^Co source (2.80 Gy/min; Gammacell 200; Atomic Energy of Canada, Mississauga, Ontario, Canada) or a ^137^Cs laboratory irradiator (0.81 Gy/min; IBL 637, CIS Bio International). Cells were irradiated with a daily dose of 2 Gy from Monday till Friday for up to 6 weeks, i.e., a maximum of 30 × 2 Gy). Culture medium was refreshed every Monday. At the end of each treatment week, culture medium was collected and cells were harvested and stored at − 80 °C until further analysis. All experiments were performed in triplicate unless indicated otherwise.

Irradiation of xenograft HT29 tumor in nude mice were carried out as published previously [12]. In brief, 5 × 10^6^ HT29 cells in 100 μL Matrigel/DMEM suspension were injected subcutaneously in the lower right flank of 6- to 7-week-old female BALB/c nude mice. Tumor growth was monitored 3–4 times per week measuring the tumor length (L), width (W), and height (H) with calipers. Tumor volume was calculated as 1/6*π*L*W*H. When the average tumor size reached a volume of approximately 100 mm^3^, the mice were randomized into experimental groups. Irradiated mice received daily 2 Gy fractions from Monday to Friday using an Xstrahl RS320 X-Ray irradiator (Xstrahl Ltd. UK). For this, mice were anesthesized by i.p. injection of 100 μL 1:1:8 hypnorm: hypnovel: sterile water after which they were placed in ±12 mm thick lead tubes with only the tumor exposed for irradiation. Following treatment, tumor tissues were collected, snap frozen and stored at − 80 °C until further analysis.

### Patient material

Snap frozen and formalin-fixed paraffin embedded primary tumor biopsies from esophageal cancer patients receiving neoadjuvant chemoradiation (paclitaxel, carboplatin and concurrent radiotherapy of 41.4 Gy in 23 fractions) were collected via endoscopy as part of an IRB-approved clinical trial (NCT02072720, METC-VUmc identifier 2013.340). Tumor biopsies were collected at baseline and during treatment, either after 1, 2, 3 or 4 weeks depending on the study cohort. Histology from all obtained biopsies was assessed by an expert pathologist (NvG). Pre-treatment biopsies were included when tumor cell percentage was > 20%. As during treatment samples could be extensively affected by radiotherapy induced tumor necrosis, accurate assessment of tumor cell was not feasible. Instead, these biopsies were obtained with extra care from a representative area on the tumor border by an expert gastroenterologist.

### RNA extraction and qPCR

RNA isolation from mouse xenografts tumors and cultured cells for RNA sequencing analysis was performed using the mirVANA kit (Life technologies), excluding the purifying miRNA step. For all other RNA isolations, TRIzol (Invitrogen) was used according to the supplier’s protocol, using chloroform for phase separation and isopropanol to precipitate the RNA. The final RNA concentration was determined using the Nanodrop ND-1000. Subsequent reverse transcription was performed on 1 μg RNA using the iScript kit (Biorad) following the suppliers’ protocol. cDNA was stored at − 20 °C until further use. qPCR was performed using 1x SYBR green supermix (Biorad), 1.5 μL cDNA and 400 nM primers in a total sample volume of 25 μL. For normalization, the primers targeting reference genes β-actin (F: TTCCTATGTGGGCGACGAG R: TCCTCGGGAGCCACACG), HPRT (F: TGCTGAGGATTTGGAAAGG R: TCACATCTCGAGCAAGACGT) and cyclo-A (F: AGCATGTGGTGTTTGGCAAA R: TCGAGTTGTCCACAGTCAGC) were used unless stated otherwise. All other primer sequences are listed in Supplementary Table 1. qPCR was performed in a CFX96 cycler (Biorad) and the following cycling conditions were used: 95 °C for 5 min, followed by 40 cycles of 95 °C for 10 s and 60 °C for 30 s, after which standard meltcurve analysis was performed.

### mRNA sequencing and data analysis

Approximately 1 μg total RNA was normalized and enriched using the NEBNext PolyA mRNA Magnetic Isolation Module (New England Biolabs) with a final elution in 18 μL, to feed into the NEBNext mRNA Library Prep Master Mix Set for Illumina (New England Biolabs) with the following modifications: post fragmentation purification was a RNA Clean Ampure XP (Agencourt) magnetic bead clean-up (2.8x volume) with 3 × 80% ethanol washes and a final elution in 15 μL buffer EB (QIAGEN). The first strand reverse transcription was conducted following protocol, but with the addition of Actinomycin D (0.05 μg/μl final concentration). The second strand reverse transcription followed the E7490 protocol, but the reaction buffer was replaced with NEBNext® Second Strand Synthesis (dNTP-free) Reaction Buffer (New England Biolabs) and a dNTP mix containing A,C,G,U at 0.3 mM for each final concentration. Double strand cDNA purification was done using Ampure XP magnetic bead clean-up (1.2x volume). End repair, A-tailing and adapter ligation were conducted following protocol with 1.8x volume Ampure XP clean-ups between steps. The PCR amplification was performed following protocol with 2 μL H2O being replaced with 2 μL USER enzyme and the Phusion polymerase being added after a 37 °C incubation for 30 min. A subsequent 12 cycles of PCR were performed using custom PCR primers [13]. Post-PCR libraries were quantified with Picogreen (Invitrogen) and size range determined using the Tapestation D1K (Agilent). Libraries were pooled equimolarly with a final quantification by qPCR before sequencing. Then, quality control was performed using FASTQC version 0.11.2. Subsequently, data filtering such as removal of technical sequences (e.g. adaptors), duplicate reads, and secondary reads were performed using Prof. Buffa’s laboratory pipelines. Quality control task was performed again after the data filtering procedures to double confirm the quality. The clean short reads were aligned to human reference genome GRCH37 using tophat2 version 2.0.13. The library type in tophat2 was set to fr-firststrand, which specified the right-most end of fragment is the first sequenced. The expected inner distance between mate pairs is set to --mate-inner-dist = 90. After that, the differential expression of each gene was estimated by cuffdiff version 2.2.1. The setting of library type is fr-firststrand, which is the same with the setting in tophat2. In the end, the consistently up-regulated genes and down-regulated genes, based on statistics of rank product, among samples are generated. The R library of Rank Product is version 2.40.0. The *p*-value and the probability of false positive of gene rank were estimated by a resampling technique with 100 random permutations.

### Microarray gene expression and data analysis

High-density oligonucleotide Expression BeadChips (Human HT12_V4, Illumina) were used for whole Genome-Wide gene expression profiling, for 3 to 4 biological replicates. In brief, 500 ng of total RNAs were reverse transcribed to synthesize first- and second- strand cDNA, purified and in vitro transcribed to synthesize biotin-labeled cRNA using the Illumina TotalPrep-96 RNA Amplification Kit (Ambion). A total of 1500 ng of biotin-labeled cRNA was then hybridized to the BeadChips at 55 °C for 18 h. The hybridized BeadChip was washed and stained with streptavidin-Cy3 according to the manufacture protocols using Illumina whole-genome gene expression direct hybridization assay (Illumina). GenomeStudio Data Analysis Software was used to visualize and analyze images generated. The Illumina microarrays were pre-processed using R package LIMMA (v3.16.8). Briefly, background correction was performed using negative controls, followed by quantile normalization and log2 transformation. Any probes whereby all samples had detection *p*-value ≥0.05 were regarded as not-expressed and subsequently removed from the dataset. Paired analysis was performed, as at least 3 matched samples were available in each group. Gene ontology enrichment analyses of differentially expressed genes were conducted using R package GOstats (v2.26.0). All visualizations and statistical analyses were performed in R statistical environment (v4.0.2).

### Elisa

Enzyme-linked immunosorbent assays were performed according to the manufacturer’s instructions (R&D systems, Abingdon, UK). Expression levels were normalized to the number of cells for the in vitro experiments and to the total protein level for the tumor xenografts.

### Clonogenic survival assay

Clonogenic survival assays were determined as described before [11]. In brief, cells were collected at different time points during the treatment period and 10.000 to 100.000 cells were plated in duplicate in T25 culture flasks and cells were grown for 14 days under normal culture conditions. At the end of each experiment, cells were fixed with 100% ethanol for 30 min, and stained with Giemsa solution (Merck, Darmstadt, Germany). Colonies (> 50 cells) were counted visually and plating efficiency (PE) was calculated by dividing the number of colonies counted by the number of cells plated. Surviving fractions (SF) were calculated by dividing the PE of irradiated cells by the PE of the non-irradiated controls.

### Statistical analysis

For the statistical analyses of mRNA sequencing and micro-array studies, please refer to the specific method description. Differences in mRNA and protein expression were tested for statistical significance with either the non-parametrical Wilcoxon signed rank test or Mann-Whitney U test for comparison of paired or independent observations in 2 groups, respectively. For multiple groups or time-course comparisons a Kruskal-Wallis test with Bonferroni post-hoc test was performed. For the comparison of HT29 xenograft tumor volumes a 2-way ANOVA with Bonferroni post-hoc test was performed. A *p*-value ≤0.05 was considered as statistically significant. Statistical analyses were performed using GraphPad Prism 8.0.0, GraphPad Software, San Diego, California US.

## Results

**Fig. 1:**
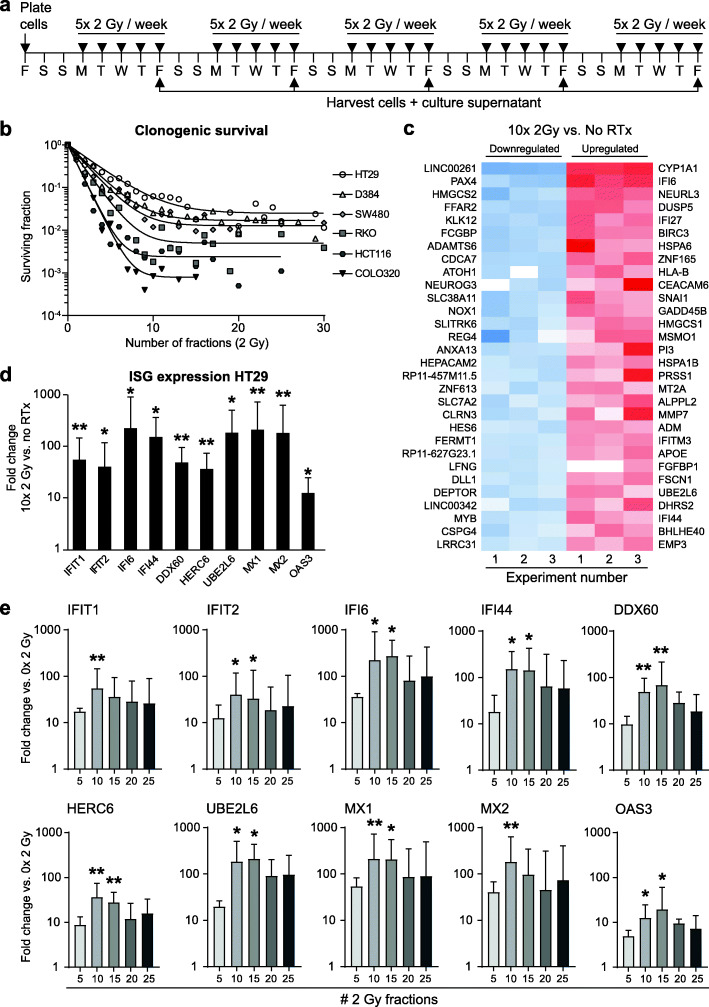
Induction of a type I IFN response in cancer cells peaks after 2 weeks of fractionated irradiation in vitro*.* Fractionated irradiation induces a type I IFN response in cancer cells*,* which peaks after 2 weeks and coincides with a convergence in clonogenic survival to a steady state. **a** Scheme of fractionated irradiation applied to human cancer cells in vitro. **b** Clonogenic survival analyses show a log-linear decline in survival during the first 2 weeks of treatment after which a steady-state survival is reached up to 6 weeks of treatment. Adapted from Van den Berg et al. [11]. **c** Heat map showing the 30 most downregulated and upregulated genes after 2 weeks of treatment vs. untreated as determined by RNA deep-sequencing of HT29 cells (*n* = 3). **d** The mRNA expression induction of a panel of 10 IFN-stimulated genes (ISGs) after 2 weeks of treatment was confirmed by qPCR (n = 3). Geometric mean + SD is shown. * *p*-value ≤0.05 vs. no radiotherapy (RTx). **e** Time course analysis of ISG mRNA expression induction shows a peak starting around 2 weeks of treatment (n = 3). Geometric mean + SD is shown. * *p*-value ≤0.05 vs 0 × 2 Gy

To identify the molecular mechanism(s) involved in the response to conventional fractionated irradiation, we set out to compare gene expression profiles in irradiated vs. non-irradiated cells. For this, HT29 colorectal carcinoma cells were subjected to a common clinically applied treatment schedule of daily 2 Gy irradiation, 5 days per week for up to 5 weeks (Fig. 1a). Since our previous work showed that clonogenic survival converges to a steady state after 2 weeks of treatment, i.e. 10 fractions (Fig. 1b), we first compared the expression at that timepoint with the expression in non-irradiated cells, cultured identically for 2 weeks. Gene expression analysis by RNA sequencing identified over a thousand differentially expressed genes (adjusted *p*-value ≤0.05) in irradiated vs. non-irradiated cells (Fig. 1c and Supplementary Tables 2 + 3). Gene ontology (GO) analysis revealed over 250 significantly enriched upregulated biological processes, amongst which the ‘type I IFN-mediated signaling pathway’ was identified as the most significantly enriched biological process (adjusted *p*-value ≤0.0001) (Supplementary Fig. S1a and Supplementary Table 4). Other identified GO terms were closely related to biological processes such as positive regulation of cell migration, angiogenesis, negative regulation of cell proliferation, amine metabolism, response to virus and nucleosome assembly. Additionally, over a hundred significantly enriched downregulated biological processes were identified, mainly related to translational processes and cell cycle (Supplementary Fig. S1b). Given the current insights in radiotherapy-induced type I interferon signaling [14, 15], as well as previous (pre) clinical trials on the combination of radiotherapy with type I interferons in cancer [16], we further focused our research on this particular response.

The induction of the type I IFN response in HT29 cells could be confirmed by qPCR with a panel of 10 interferon stimulated genes (ISGs) that are linked to this response (Fig. 1d). Moreover, the increased expression of ISGs by 10 × 2 Gy irradiation could be confirmed in multiple cancer cell lines, including high-grade astrocytoma cells (D384) and different colorectal cancer cell lines (SW480, HCT116, COLO320, RKO; Supplementary Fig. S2). To determine the dynamics of the response, the ISG expression was analyzed weekly for up to 5 weeks. This showed a slight induction in expression for most ISGs after 5 fractions, and a peak induction after 2 to 3 weeks of treatment (Fig. 1e and Supplementary Fig. S3a). Continuation of RTx eventually resulted in a decreased expression, although it generally remained above the level of non-irradiated cells. Of note, while single dose irradiation also induced dose-dependent ISG expression, this typically leveled off after 6 Gy (Supplementary Fig. S3b). Collectively, these data show that fractionated RTx induces an intrinsic type I interferon response in vitro which peaks within 2 to 3 weeks of treatment and coincides with the development of a steady state in clonogenic survival.

**Fig. 2:**
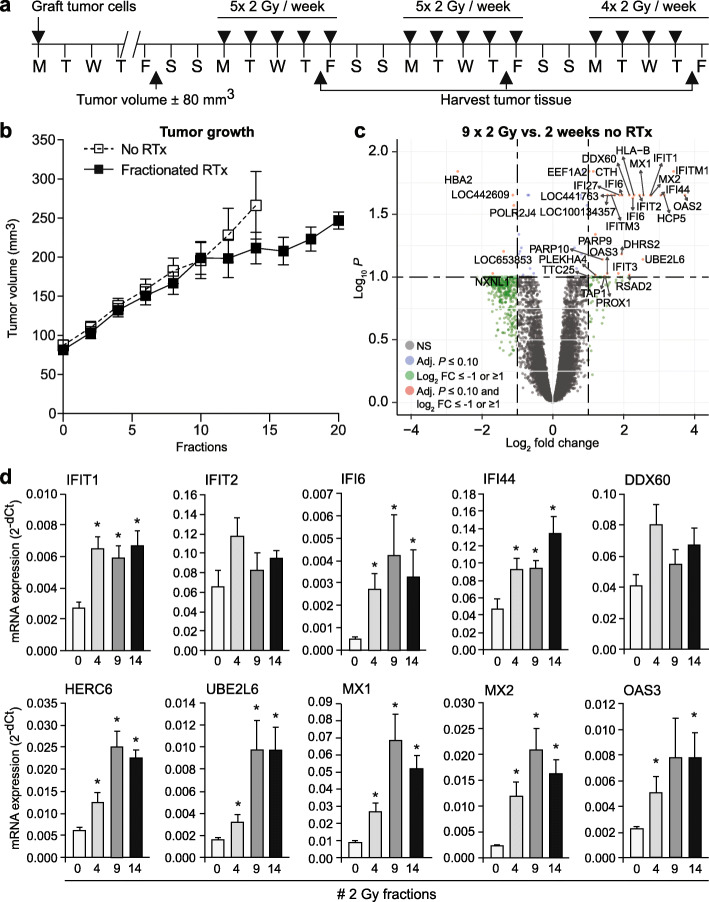
Induction of a type I IFN response in tumor tissue peaks after 2 weeks of fractionated irradiation in vivo*.* The induction of a type I IFN response upon fractionated radiotherapy is confirmed in a HT29 xenograft model. **a** Scheme of fractionated irradiation applied to HT29 xenograft tumor in mice. **b** Tumor growth curves of HT29 xenograft tumors with (black squares) or without (white squares) irradiation. Note the growth delay starts around day 10 and recovers around day 17 (*n* = 5 mice/group). **c** Volcano plot of microarray data comparing gene expression in HT29 xenograft tumors after 2 weeks of RTx vs. no radiotherapy (RTx). NS = not significant. FC = fold change. **d** Time course analysis of ISG mRNA expression induction shows a gradual increase that peaks around 2 weeks of treatment. * *p*-value ≤0.05 vs. 0 × 2 Gy

To extend these findings, HT29 xenograft tumors were locally irradiated using the same clinical schedule as the cultured cells, i.e., 2 Gy per day, 5 days per week for up to 3 weeks (Fig. 2a). Tumor growth showed a delay after 2 weeks of treatment but appeared to recover in week 3 (Fig. 2b and Supplementary Fig. S4a). Next, gene expression profiles of non-irradiated tumors vs. tumors that received 1, 2 and 3 weeks of radiotherapy were obtained using human microarray analysis. After 2 weeks of treatment, 34 differentially expressed genes in irradiated vs. non-irradiated tumor tissues were identified, of which 5 showed decreased expression and 29 showed increased expression (Fig. 2c, Supplementary Table 5). Gene ontology analysis revealed 52 significantly enriched biological processes, amongst which the ‘type I IFN-mediated signaling pathway’ was again identified as the most significantly enriched pathway (*p*-value ≤0.0001, count 18/61) (Supplementary Fig. S4b and Supplementary Table 6). Interestingly, a less pronounced but similar gene expression profile was observed after 1 and 3 weeks of irradiation, whereas a single dose of 5 Gy resulted in more differentially expressed genes (Supplementary Fig. S4c**)**. Expression analysis of the same ISG signature panel as used before, again confirmed the induction of a type I IFN response (Supplementary Fig. S4d). Moreover, in line with our observations in the cell lines, time course analysis revealed that the expression of the ISGs peaked after 2 to 3 weeks of treatment (Fig. 2d). Altogether, these results show that fractionated RTx induces a potent type I interferon response in tumor cells after 2 to 3 weeks of treatment.

**Fig. 3:**
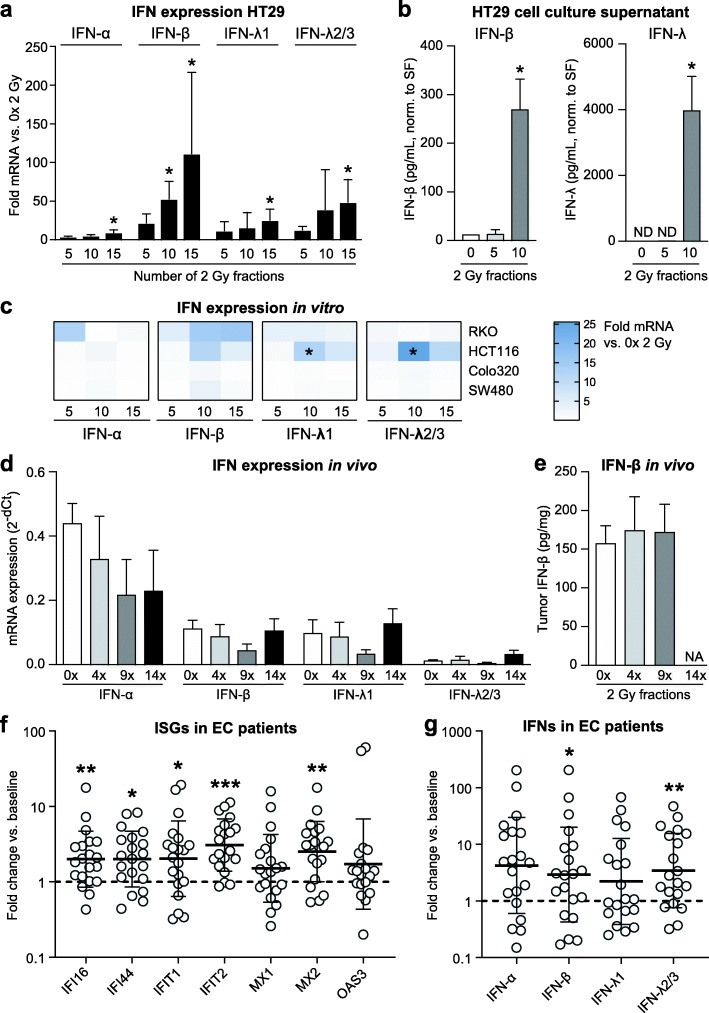
Patterns of type I and III interferon induction upon fractionated irradiation. Different patterns of either type I and/or type III interferon induction occur in vitro*,* in vivo and patients with esophageal cancer during the course of fractionated radiotherapy, independent of ISG induction. **a** mRNA expression analyses of interferon expression in HT29 cells during fractionated irradiation (*n* = 3). * *p*-value ≤0.05 vs. 0 × 2 Gy. **b** Levels of IFN-β and IFN-λ protein in cell culture supernatants of HT29 cells during fractionated irradiation (n = 3). * *p*-value ≤0.05 vs. 0 × 2 Gy. CM = culture medium. SF = surviving fraction. **c** mRNA expression analyses of interferon expression in RKO, HCT116, COLO320 and SW480 cells during fractionated irradiation vs. 0 × 2 Gy. * *p*-value ≤0.05 vs. 0 × 2 Gy. **d** mRNA expression analyses of interferon expression in HT29 xenograft tumors during fractionated irradiation (n = 5 mice/group). **e** Levels of IFN-β and IFN-λ protein in mouse serum during fractionated irradiation (n = 5 mice/group). **f** mRNA expression levels of ISG expression in patient-matched tumor samples from esophageal cancer patients (*n* = 20) prior to or during chemoradiotherapy. Fold expression in on-treatment samples vs. pre-treatment is shown. * *p*-value ≤0.05 vs. matched pre-treatment samples. **g** Similar as in (**f**) for fold change in mRNA expression levels of different IFNs. * *p*-value ≤0.05 vs. matched pre-treatment samples

To determine which type I IFN could have triggered the response, we analyzed the mRNA expression of two key family members in vitro, i.e., IFN alpha (*IFN-α*) and IFN beta (*IFN-β*). Since type III interferons (IFN lambda; IFN-λ) were recently shown to be induced by RTx in HT29 [17], these cells were included as a positive control. Analysis of fractionally irradiated HT29 tumor cells revealed that the treatment predominantly induced the mRNA expression and protein secretion of IFN-β and IFN-λ (Fig. 3a+b). Other cell lines subjected to fractionated irradiation displayed either a modest increase in mRNA expression of either *IFN-α*, *IFN-β*, *IFN-*λ or a combination (HCT116 and RKO), or no interferon induction at all (SW480 and Colo320) (Fig. 3c). Interestingly, all of these cell lines showed clear induction of ISG expression in response to irradiation, albeit less profound in the cell lines lacking interferon expression (Supplementary Fig. S2). In the xenograft tumors, no changes in the expression of any of the different interferons could be detected (Fig. 3d+e). These findings suggest an uncoupling between the induction of ISGs and the expression of interferons, the latter usually mediating ISG expression. Of note, all the cell lines expressed the appropriate IFN receptors required to be responsive to the different IFNs (Supplementary Fig. S5).

**Fig. 4:**
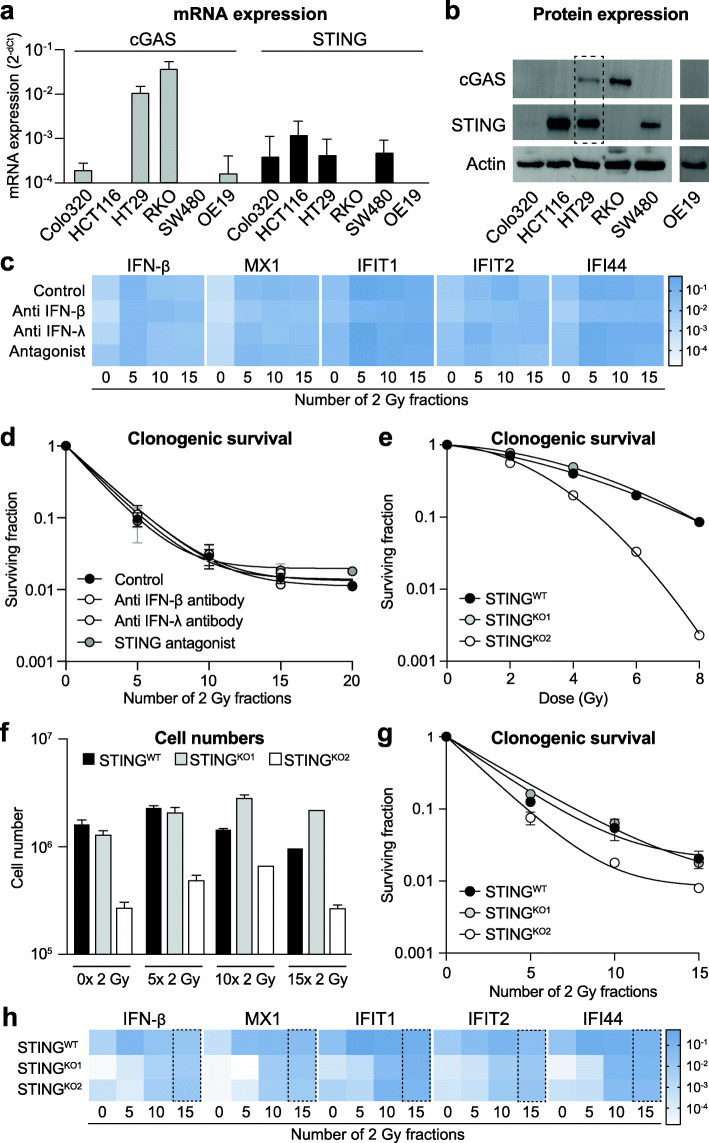
ISG induction upon fractionated irradiation occurs independent of STING, IFN-β or IFN-λ. Interferon stimulated genes (ISGs) can be induced independent of the interferons known to mediate this response, or the upstream regulator protein STING. **a** mRNA expression levels of cGAS (grey bars) and STING (black bars) in different cancer cell lines. **b** Western blots showing protein expression of cGAS and STING in different cancer cell lines. Actin staining was used as loading control. The dotted box shows the only cell line, i.e. HT29, in which both cGAS and STING protein expression could be detected. **c** Heat map of mRNA expression of different ISGs and IFN-β in HT29 cells treated with fractionated irradiation in the presence or absence of either anti IFN-β antibody, anti IFN-λ antibody or a STING antagonist. No significant changes were observed in the presence of any of the treatments as compared to irradiation alone (n = 3). **d** Clonogenic survival of HT29 during fractionated irradiation in the presence or absence of either anti IFN-β antibody, anti IFN-λ antibody or a STING antagonist. No significant changes in surviving fractions were observed in the presence of any of the latter treatments as compared to irradiation alone (n = 3). **e** Clonogenic survival of HT29 wild-type cells and two HT29 STING knockout cells in response to single dose irradiation. STING^KO2^ shows higher radiosensitivity as compared to wild type cells. **f** Cell numbers of HT29 wild-type cells and two HT29 STING knockout cells during fractionated irradiation. While STING^KO2^ displayed slower growth already at base-line, fractionated irradiation did not affect growth of knockout cells compared to wild type cells. **g** Clonogenic survival of HT29 wild-type cells and two HT29 STING knockout cells during fractionated irradiation. STING^KO2^ shows higher radiosensitivity as compared to wild type cells. h) Heat map of mRNA expression of different ISGs and IFN-β in HT29 wild-type cells and two HT29 STING knockout cells during fractionated irradiation (n = 2). At baseline (0 × 2 Gy) both knockout cell lines show lower expression of all genes analyzed as compared to wild-type cells. At the end of the treatment period (15 × 2 Gy, dotted box) no more difference in expression levels is observed in wild-type vs. knockout cells for any of the genes analyzed

To assess the clinical relevance of these findings, we analyzed whether a type I IFN response occurs in cancer patients, in the context of a clinical pilot study (NCT02072720) in esophageal cancer patients receiving neoadjuvant chemoradiotherapy (CRT) with paclitaxel, carboplatin and concurrent radiotherapy (41.4 Gy in 23 fractions of 1.8 Gy). Tumor biopsies of 20 patients (see Supplementary Table S7 for patient characteristics) were collected at baseline and after 1, 2, 3 or 4 weeks of treatment, in successive cohorts. Subsequent expression analysis revealed that expression levels of 5 out of 7 investigated ISGs were significantly elevated during treatment as compared to baseline (Fig. 3f**)**. Of note, while the number of patients in this small pilot study did not allow us to confirm an association between pre-treatment ISG expression levels and response to treatment [18, 19], we did observe ISG expression levels were highest in patients that had received 2 weeks of treatment (Supplementary Fig. S6a). The latter is in line with our findings in tumor cells and xenograft tumors. Furthermore, a modest induction of all interferons was seen (significant for IFN-β and IFN−λ2/3; Fig. 4g), but again ISG expression appeared to occur independent of type I interferons, as only a weak correlation was observed between induction of IFN-β and 3 out of 7 ISGs (Supplementary Fig. S6b). Thus, both in a preclinical immunocompromised xenograft model as well as in a clinical setting, commonly applied fractionated irradiation triggers a type I interferon response independent of actual type I interferon expression induction.

Since the induction of a type I interferon response during radiotherapy has been linked to cGAS/STING signaling [14, 20, 21], we further evaluated the role of STING as well as of interferon expression on the induction of ISGs during fractionated irradiation. Analysis of both mRNA and protein expression showed low or even absent basal expression of cGAS, STING or both in the majority of cell lines, except for HT29 (Fig. 4a+b). Since all cell lines did show elevated ISG expression during fractionated irradiation, these findings suggest that the radiation-induced type I interferon response does not depend on cGAS/STING signaling. Of note, when cells that were deficient in either cGAS or STING were irradiated, the expression of the absent proteins was not induced (Supplementary Fig. S7a).

To further study the disconnection between the radiation-induced type I interferon response and cGAS/STING activation or type I interferon expression, we irradiated HT29 cells (which express all components of the pathway) in the presence of either anti-IFN-β antibody, anti-IFN-λ antibody or a STING antagonist. Optimal antibody treatment conditions were based on literature [16] and the levels of IFN-β and IFN-λ in cell culture supernatants. In addition, direct effects of treatment on cell viability were excluded (Supplementary Fig. S7b). Also, the inhibitory function of the STING antagonist was confirmed by Western blot showing reduced phosphorylation of the downstream target protein Tank Binding Kinase (pTBK) after 4 Gy irradiation as compared to no irradiation (Supplementary Fig. S7c). In line with our previous observations, neither treatment with anti-IFN antibodies nor treatment with the STING antagonist had any effect on the induction of ISG or IFN expression during fractionated irradiation (Fig. 4c). Moreover, neither treatment affected the clonogenic survival of HT29 cells prior to irradiation (Supplementary Fig. S7d) or during fractionated irradiation (Fig. 5 d). This was not due to lack of treatment efficacy, since anti-IFNβ antibody treatment did neutralize the known inhibitory effect of IFN-β on cell growth (Supplementary Fig. S7e). Again, these data suggest that the type I IFN response that is triggered by fractionated irradiation occurs independent of cGAS/STING signaling or induction of IFN expression.

Since our findings are different from the previously published role of STING in the response to radiotherapy and could be due to minimal undetected levels of STING, we also generated HT29 STING knockout cells using CRISPR/Cas gene editing. In 8 out of 10 single cell clones, knockdown could be confirmed by Western Blot (Supplementary Fig. S8a). Two clones were selected for further analysis and DNA sequencing confirmed gene editing at the expected location in exon 6, causing a frameshift with two adjacent premature stop codons (Supplementary Fig. S8b). Interestingly, one of the STING knockout clones showed a phenotype similar to the wild type cells while the other knockout clone showed reduced cell growth (data not shown) and increased radiosensitivity (Fig. 4e). This clonal difference in growth and radiosensitivity was further illustrated when both clones were subjected to fractionated irradiation for 3 weeks (Fig. 4f+g). Despite these differences in phenotype, both knockout cell lines showed a similar, albeit delayed, induction of ISG expression as compared to the wild type cells (Fig. 4h). The latter suggests that STING, while it contributes to the induction of a type I IFN response during fractionated irradiation, is not essential for this response to occur. Altogether, STING, IFN-β as well as IFN-λ appear to be dispensable for activation of a type I IFN response during fractionated irradiation.

## Discussions

In this study we demonstrate that a commonly applied clinical schedule of conventional low-dose fractionated irradiation (daily fractions of 2 Gy for 5 days per week, up to 6 weeks) induces an intrinsic type I interferon (IFN) response in tumor cells that is characterized by an increased expression of interferon stimulated genes (ISGs). The response peaks within 3 weeks of treatment and coincides with a convergence to a plateau in clonogenic survival in vitro and treatment resistance in vivo. It also occurs in tumor tissues from esophageal cancer patients during chemoradiotherapy. Importantly, the type I IFN response can be induced independently of a specific type I IFN or of STING-mediated signaling. Collectively, these findings suggest a potential clinical benefit of targeting specific type I interferon response genes (ISGs), irrespective of targeting STING or type I interferons during fractionated low-dose radiotherapy.

The induction of a type I IFN response by fractionated irradiation has been described previously in cancer cells of different origin. For example, using either breast cancer cells, prostate cancer cells or gliosarcoma cells Tsai et al. found significant induction of several ISGs after 5 × 2 Gy while 1 × 10 Gy did not trigger expression [9]. This was repeatedly confirmed by another group that described a more prominent induction of ISGs in prostate cancer cells after fractionated irradiation (10 × 1 Gy) as compared to single dose (1 × 10 Gy) irradiation [22–24]. More recently, Vanpouille-Box et al. reported increased expression of ISGs in different mouse and human breast cancer cells after fractionated irradiation (3 × 8 Gy), but not after single dose irradiation (1 × 20 Gy) [10]. Our current data are in line with all these in vitro findings and confirm that the response is triggered in in vivo as well, albeit to a somewhat lesser extent [9, 10]. Importantly, we show that the response becomes particularly activated after 2 weeks of radiotherapy and remains highly activated throughout the course of treatment. Together with the observation that the response is activated during fractionated radiotherapy in esophageal cancer patients, these findings suggest a potential clinical relevance of type I IFN signaling in the response to treatment. In line with this, the induction of type I interferons as well as other molecules by radiotherapy has been shown to elicit an anti-tumor immune response [4, 25]. At the same time, radiotherapy can hamper an adequate immune response which, together with the potential immune induction, has spurred interest to combine radiotherapy with immunotherapy, particular with checkpoint inhibitors [4, 25, 26]. Regarding the direct combination of radiotherapy with type I IFNs, the outcomes of clinical trials have been ambiguous and increased toxicity frequently led to negative recommendations on this treatment approach [16]. Possibly, this is related to inadequate dose-scheduling of both treatment modalities as radiation dose and scheduling have been shown to affect the immunostimulatory activity [26]. Our current findings indeed suggest that there is no rational for prolonged administration of IFNs or STING agonists during radiotherapy, particularly if this results in increased toxicity. At the same time, to boost anti-tumor immune responses, IFNs or STING-agonists might be beneficial but only when administered briefly, i.e., in the first weeks of fractionated radiotherapy. Future studies should thus focus on optimal dose-scheduling of radiotherapy in combination with type I IFN-targeted treatment.

Apart from therapeutic options, the observed type I IFN expression signature could also have diagnostic/prognostic value. Previously, an IFN-related DNA damage resistance signature (IRDS) has been found to be predictive for poor survival outcome in GBM patients [18] as well as for the efficacy of adjuvant chemotherapy and local-regional control after radiation in breast cancer patients [19]. Although we could not confirm the latter in our patient series due to the small sample size, it can be speculated that the observed induction of a type I IFN response in our patient group during (chemo) radiotherapy serves as a radioprotective mechanism. This is supported by our finding that the peak in ISG expression after 2 weeks of irradiation coincides with the convergence to a plateau in clonogenic survival [11]. On the other hand, Guggenberger et al. described that 4 weeks of fractionated low-dose irradiation (daily dose of 0.5 or 1.0 Gy) of primary cultures of benign prostate epithelial cells resulted in downregulation of a type I interferon expression signature [27]. Interestingly, the expression analyses in that study were performed 1 week after completion of the fractionated irradiation schedule. Apart from differences in fraction dose and cell type, this difference in timing of expression analysis most likely accounts for the discrepancy between both observations. In fact, a normalization or downregulation of the IFN response in the days or weeks after therapy supports our previous observation that fractionated irradiation induces transient and reversible radioresistance rather than acquired radioresistance [11]. This reversal or ‘normalization’ of the response should be further investigated, especially in the context of clinical samples, as it could provide therapeutic opportunities.

Although we observed that fractionated irradiation consistently induced expression signatures that are characteristic of a type I IFN response, there was no clear association with the expression of a specific type I IFN. Generally, antigen-presenting cells are considered as the main source of type I IFNs although intrinsic cancer cell production has been demonstrated after anthracycline-based chemotherapy [28] and radiotherapy [10]. In line with this, we did observe that irradiation can induce IFN-β or IFN-λ expression in cancer cells [17, 20, 29]. However, the induction did not occur in all cell lines even though downstream ISG expression was always triggered. Furthermore, blocking either IFN-β or IFN-λ did not affect the induction of ISGs during fractionated irradiation. This contradicts studies that have shown that the induction of a type I IFN response does not occur in cells that lack IFNAR1 (Interferon Alpha and Beta Receptor Subunit 1) expression. Since we did not block IFNAR1 or any of the other IFN receptors, we cannot rule out that other IFN family members might be responsible for the induction of ISG expression. While this could be further explored, our data still indicate that neither IFN-β nor IFN-λ are required for the induction of a type I IFN response during fractionated irradiation.

Of note, a mechanism that has been proposed to prevent the induction of the type I IFN response involves induction of the DNA exonuclease Three Prime Repair Exonuclease 1 (TREX1) [10]. This protein was found to degrade cytosolic DNA that accumulates in irradiated cancer cells, thereby impeding a type I IFN response [10]. It has been shown that this mechanism is triggered after single high dose (> 12–18 Gy) irradiation and not after fractionated irradiation (3 × 8 Gy) [10]. Interestingly, Erdal et al. linked accumulation of cytosolic DNA in TREX1-deficient human breast cancer cells and mouse embryonic fibroblast cells to increased radioresistance and ISG signaling after a single dose of 6 to 10 Gy. Disrupting the downstream transcription factor interferon regulatory factor 3 (IRF-3) in these cells completely abolished ISG induction and the observed radioresistance. Although we cannot rule out involvement of TREX1 in cancer cells that do not show increased IFN expression after fractionated irradiation with 2 Gy (Colo320 and SW480), these cells still showed induction of ISG expression and a steady state in clonogenic survival [11].

The disconnection between specific type I or type III IFN expression and the induction of ISG expression is further supported by our finding that the response also occurs in cancer cells that lack either cGAS (cyclic GMP-AMP Synthase) or STING (Stimulator of interferon genes) expression. It has been shown that the cGAS/STING signaling axis is a key regulator of the innate immune response [21, 30, 31]. This pathway also triggers type I IFN expression in response to DNA damage [10, 14, 17, 29]. Indeed, several studies have described that inhibition or complete knockout of either cGAS or STING hampers an adequate type I IFN response in vitro and in vivo [10, 14, 17, 29, 32]. In contrast, we observed comparable induction of ISGs during fractionated irradiation of cancer cell lines, irrespective of cGAS or STING expression. Also, treatment with a STING antagonist or knockout of STING could not prevent ISG expression induction. However, we did observe a delay in expression induction in the absence of STING in HT29 STING-knockout cells. Apparently, STING facilitates or contributes to the activation of ISG expression during the early phase of fractionated irradiation, but it is not indispensable. Our observation also implies that other pathways contribute to the activation of ISG expression. In that regard, pattern-recognition receptors, such as Toll-like receptors, (TLRs) are known to activate innate type I IFN signaling [33] and agonists of TLRs can improve the response to radiotherapy [34, 35]. On the other hand, it has previously been demonstrated that key downstream adapter molecules of TLR signaling, like Myeloid Differentiation primary-response protein 88 (MyD88) and TIR-domain-containing adaptor protein inducing IFN-β (TRIF), are not essential to induce a type I IFN response during radiotherapy [14]. Interestingly, RNA activated innate immune pathways controlled by RIG-I (retinoic acid-inducible gene I) and MDA5 (melanoma differentiation-associated protein 5) are significantly less affected by loss-of-function mutations or epigenetic silencing as compared to STING [36] and can be linked type I IFN signaling in a STING-independent manner. RIG-I and MDA-5, triggered by either RNA from dying neighboring cells or production of small cytosolic RNA fragments via RNA Polymerase III [37, 38], could elicit a similar set of transcription factors involved in expression of type I IFNs via Mitochondrial Antiviral-Signaling protein (MAVS). Also, other cytosolic DNA sensors, e.g. IFI16 and DDX41, might play a role as extensively reviewed recently [39]. The interplay between such cytosolic DNA/RNA sensing mechanisms in controlling type I IFN signaling during (fractionated) irradiation should be further studied.

Finally, the exact role of type I IFN signaling in the response to radiotherapy should be further explored. As recently reviewed by us and others, type I IFNs can exert both intrinsic and extrinsic anti-tumor effects. This includes inhibition of cell growth and migration, induction of apoptosis and senescence, and activation of T-cell mediated immunity [16, 40]. As such, the induction of a type I IFN response during fractionated irradiation could be considered as beneficial. At the same time, as described above, combining type I IFN treatment with radiation therapy in the clinic has been met with increased toxicity and limited or no clinical benefit [16]. In addition, high tumoral expression of ISGs or upstream transcription factors like STAT1 (Signal Transducer and Activator of Transcription 1) are indicators of radioresistance and a predictor of poor patient survival [18, 19, 41, 42]. We also observed an elevated type I IFN response in cancer cells at the time they adopt a radiotolerant phenotype. Thus, activation of type I IFN signaling by DNA damaging agents like radiotherapy might not be beneficial at all [43].

## Conclusions

In conclusion, our current findings indicate that clinically applied fractionated low-dose irradiation triggers expression of type I IFN stimulated genes independent of interferons or STING-mediated signaling. While the exact underlying mechanism needs to be resolved, our data provide novel insights in the relevance of the type I interferon response and STING signaling during low-dose fractionated irradiation which are relevant for current efforts that aim to target this response in the context of combination radiotherapy. In particular, the timing of type I IFN-targeted treatment during radiotherapy should be carefully explored as it might induce beneficial and detrimental effects depending upon a delicate balance between responses within the tumor microenvironment, including but not restricted to STING signaling pathway capacity.

## Supplementary Information

**Additional file 1.**

**Additional file 2.**

## Data Availability

The datasets used and/or analyzed during the current study are available from the corresponding author on reasonable request.

## Abbreviations

cGAS: Cyclic GMP-AMP Synthase
CRT: Chemoradiotherapy
DDX41: DEAD-box helicase 41
DMEM: Dulbecco’s Modified Eagle Medium
GBM: Glioblastoma Multiforme
GO: Gene Ontology
IFI16: Interferon gamma Inducible protein 16
IFN: Interferon
IFN-α: Interferon alpha
IFN-β: Interferon beta
IFN-λ: Interferon lambda
IFNAR1: Interferon Alpha and Beta Receptor Subunit 1
IRF-3: Interferon Regulatory Factor 3
ISG: Interferon Stimulated Gene
MAVS: Mitochondrial Antiviral-Signaling protein
MDA5: Melanoma Differentiation-Associated protein 5
pTBK: phosphorylated Tank Binding Kinase
RIG-I: Retinoic acid-Inducible Gene I
RTx: Radiotherapy
STAT1: Signal Transducer and Activator of Transcription 1
STING: Stimulator of interferon genes
TREX1: Three prime Repair Exonuclease 1
