# Supplementary material for: Interferon- and STING-independent induction of type I interferon stimulated genes during fractionated irradiation
Source: J Exp Clin Cancer Res. 2021 May 8;40:161. doi: 10.1186/s13046-021-01962-2 (PMC8106844; doi:10.1186/s13046-021-01962-2)
Supplement: Supplementary file 1 — Additional file 1. [file 13046_2021_1962_MOESM1_ESM.docx]

**Supplementary tables**

**Supplementary Table 1.** Primer sequences.

| **Gene (human)** | **Forward primer (5'-3')** | **Reverse primer (5'-3')** |
| --- | --- | --- |
| DDX60 | GTTTCTTGGAAGAGAGTTACCC | GACCTTCCTTGCCCAAGAATG |
| IFI6v2 | CAGGTGAGAATGCGGGTAAG | ATCGCAGACCAGCTCATCAG |
| IFI44 | ACAGATGTTGTAATCAAGGGCC | GGTGTACATAGTCCTAGTTTCC |
| UBE2L6v2 | CGTCTCCGCACAAAGACC | GCAGGTTGAAGGCTTTCAGG |
| HERC6 | ACAAAGCTAACTGTCGACTACC | ACTGAAAATAACAGGACTGGG |
| INF alpha pan | CCATCYCTGYCYTCCATGAG | GATTTCTGCTCTGACAACCTCC |
| IFN beta | AAACTCATGAGCAGTCTGCA | AGGAGATCTTCAGTTTCGGAGG |
| IFN lambda 1 | CGCCTTGGAAGAGTCACTCA | GAAGCCTCAGGTCCCAATTC |
| IFN lambda 2/3 | GCCACATAGCCCAGTTCAAG | TCCTTCAGCAGAAGCGACTC |
| IFIT1 | ATGGTGATGTCATCAGGTCAAG | CACACTGTATTTGGTGTCTAGG |
| IFIT2 | CAATAGCAAGCTACCGTC | GAACATCTGTTACACCTGG |
| MX1 | CCATATTTCAGGGATCTGC | GCTCCTCTGTTATTCTCTG |
| MX2 | AGTATCGAGGCAAGGAGC | ACGTTAATGAAAGCTTGCTG |
| OAS2 | GACAACTTTGACATTGCTG | AACTGGATCCAAGATTACTG |
| OAS3 | GGTCAACTATAGCACTGAG | GATGTCCCGTCTCTACTC |

**Supplementary Table 2.** RNA-sequencing of non-irradiated versus 10x 2 Gy HT29 cells *in vitro*. List of top 50 up-regulated genes in HT29 cells *in vitro* after 10x 2 Gy, compared to non-irradiated cells (n=3). Change in expression displayed as log(2) fold change.

|  |  |  | **Log(2) fold change** | | |
| --- | --- | --- | --- | --- | --- |
| **Gene ID** | **Name** | **Symbol** | **exp 1** | **exp 2** | **exp 3** |
| ENSG00000140465 | cytochrome P450, family 1, subfamily A, polypeptide 1 | CYP1A1 | 5.9 | 5.7 | 6.4 |
| ENSG00000126709 | interferon, alpha-inducible protein 6 | IFI6 | 6.5 | 5.2 | 5.9 |
| ENSG00000163121 | neuralized E3 ubiquitin protein ligase 3 | NEURL3 | 4.4 | 4.9 | 4.2 |
| ENSG00000138166 | dual specificity phosphatase 5 | DUSP5 | 4.8 | 4.6 | 3.4 |
| ENSG00000165949 | interferon, alpha-inducible protein 27 | IFI27 | 4.9 | 3.2 | 4.1 |
| ENSG00000023445 | baculoviral IAP repeat containing 3 | BIRC3 | 4.8 | 3.6 | 3.5 |
| ENSG00000173110 | heat shock 70kDa protein 6 (HSP70B') | HSPA6 | 7.9 | 2.9 | 2.6 |
| ENSG00000197279 | zinc finger protein 165 | ZNF165 | 4.3 | 3.1 | 4.4 |
| ENSG00000234745 | major histocompatibility complex, class I, B | HLA-B | 3.2 | 4.4 | 2.6 |
| ENSG00000086548 | carcinoembryonic antigen-related cell adhesion molecule 6 | CEACAM6 | 1.5 | 2.5 | 8.5 |
| ENSG00000124216 | snail family zinc finger 1 | SNAI1 | 4.9 | 3.0 | 2.5 |
| ENSG00000099860 | growth arrest and DNA-damage-inducible, beta | GADD45B | 4.2 | 3.4 | 2.6 |
| ENSG00000112972 | 3-hydroxy-3-methylglutaryl-CoA synthase 1 (soluble) | HMGCS1 | 2.2 | 4.0 | 3.6 |
| ENSG00000052802 | methylsterol monooxygenase 1 | MSMO1 | 1.4 | 4.4 | 4.3 |
| ENSG00000124102 | peptidase inhibitor 3, skin-derived | PI3 | 1.9 | 2.2 | 6.9 |
| ENSG00000204388 | heat shock 70kDa protein 1B | HSPA1B | 3.4 | 2.6 | 3.6 |
| ENSG00000204983 | protease, serine, 1 (trypsin 1) | PRSS1 | 2.2 | 1.7 | 6.9 |
| ENSG00000125148 | metallothionein 2A | MT2A | 3.5 | 3.6 | 1.9 |
| ENSG00000163286 | alkaline phosphatase, placental-like 2 | ALPPL2 | 2.2 | 2.7 | 4.8 |
| ENSG00000137673 | matrix metallopeptidase 7 | MMP7 | 3.9 | 0.6 | 6.5 |
| ENSG00000148926 | adrenomedullin | ADM | 3.2 | 3.3 | 2.5 |
| ENSG00000142089 | interferon induced transmembrane protein 3 | IFITM3 | 3.2 | 2.6 | 3.5 |
| ENSG00000130203 | apolipoprotein E | APOE | 3.0 | 2.3 | 4.0 |
| ENSG00000137440 | fibroblast growth factor binding protein 1 | FGFBP1 | NA | NA | 3.0 |
| ENSG00000075618 | fascin homolog 1, actin-bundling protein | FSCN1 | 2.9 | 2.5 | 3.8 |
| ENSG00000156587 | ubiquitin-conjugating enzyme E2L 6 | UBE2L6 | 3.7 | 3.6 | 1.5 |
| ENSG00000100867 | dehydrogenase/reductase (SDR family) member 2 | DHRS2 | 3.1 | 1.7 | 5.0 |
| ENSG00000137965 | interferon-induced protein 44 | IFI44 | 4.1 | 2.8 | 2.1 |
| ENSG00000134107 | basic helix-loop-helix family, member e40 | BHLHE40 | 1.7 | 4.0 | 2.9 |
| ENSG00000142227 | epithelial membrane protein 3 | EMP3 | 3.3 | 2.1 | 3.7 |
| ENSG00000177469 | polymerase I and transcript release factor | PTRF | 4.3 | 1.7 | 3.4 |
| ENSG00000163659 | TCDD-inducible poly(ADP-ribose) polymerase | TIPARP | 2.7 | 3.4 | 2.5 |
| ENSG00000166831 | RNA binding protein with multiple splicing 2 | RBPMS2 | 2.6 | 3.4 | 2.5 |
| ENSG00000196352 | CD55 molecule, decay accelerating factor for complement | CD55 | 1.3 | 3.7 | 3.6 |
| ENSG00000204389 | heat shock 70kDa protein 1A | HSPA1A | 3.1 | 2.2 | 3.7 |
| ENSG00000181126 | major histocompatibility complex, class I, V (pseudogene) | HLA-P | 3.3 | 2.9 | 2.5 |
| ENSG00000171401 | keratin 13 | KRT13 | 2.7 | 1.6 | 5.3 |
| ENSG00000204103 | v-maf avian musculoaponeurotic fibrosarcoma oncogene homolog B | MAFB | 4.7 | 1.8 | 2.7 |
| ENSG00000185022 | v-maf avian musculoaponeurotic fibrosarcoma oncogene homolog F | MAFF | 3.6 | 3.4 | 1.4 |
| ENSG00000125657 | tumor necrosis factor (ligand) superfamily, member 9 | TNFSF9 | 3.6 | 3.4 | 1.4 |
| ENSG00000105388 | carcinoembryonic antigen-related cell adhesion molecule 5 | CEACAM5 | 1.4 | 2.2 | 6.3 |
| ENSG00000186480 | insulin induced gene 1 | INSIG1 | 1.1 | 4.2 | 3.1 |
| ENSG00000189325 | chromosome 6 open reading frame 222 | C6orf222 | 3.1 | 2.5 | 3.0 |
| ENSG00000164949 | GTP binding protein overexpressed in skeletal muscle | GEM | 3.3 | 3.0 | 1.9 |
| ENSG00000232810 | tumor necrosis factor | TNF | 3.6 | 2.6 | 2.2 |
| ENSG00000250606 | protease, serine, 3 pseudogene 2 | PRSS3P2 | 2.4 | 1.8 | 4.5 |
| ENSG00000156966 | UDP-GlcNAc:betaGal beta-1,3-N-acetylglucosaminyltransferase 7 | B3GNT7 | 2.1 | 2.1 | 4.3 |
| ENSG00000035862 | TIMP Metallopeptidase Inhibitor 2 | TIMP2 | 1.6 | 3.1 | 3.6 |
| ENSG00000172602 | Rho Family GTPase 1 | RND1 | 4.3 | 2.1 | 2.0 |
| ENSG00000112299 | vanin 1 | VNN1 | 3.6 | 1.8 | 3.2 |

**Supplementary Table 3.** RNA-sequencing of non-irradiated versus 10x 2 Gy HT29 cells *in vitro*. List of top 50 down-regulated genes in HT29 cells *in vitro* after 10x 2 Gy, compared to non-irradiated cells (n=3). Change in expression displayed as log(2) fold change.

|  |  |  | **Log(2) fold change** | | |
| --- | --- | --- | --- | --- | --- |
| **Gene ID** | **Name** | **Symbol** | **exp 1** | **exp 2** | **exp 3** |
| ENSG00000259974 | long intergenic non-protein coding RNA 261 | LINC00261 | -5.1 | -5.0 | -4.7 |
| ENSG00000106331 | paired box 4 | PAX4 | -3.1 | -4.0 | -4.2 |
| ENSG00000134240 | 3-hydroxy-3-methylglutaryl-CoA synthase 2 (mitochondrial) | HMGCS2 | -5.0 | -3.3 | -2.6 |
| ENSG00000126262 | free fatty acid receptor 2 | FFAR2 | -3.1 | -3.5 | -3.3 |
| ENSG00000186474 | kallikrein-related peptidase 12 | KLK12 | -3.5 | -3.0 | -3.5 |
| ENSG00000090920 | Fc fragment of IgG binding protein | FCGBP | -3.9 | -3.3 | -2.9 |
| ENSG00000049192 | ADAM metallopeptidase with thrombospondin type 1 motif, 6 | ADAMTS6 | -3.8 | -3.3 | -2.7 |
| ENSG00000144354 | cell division cycle associated 7 | CDCA7 | -3.2 | -3.2 | -3.3 |
| ENSG00000172238 | atonal homolog 1 (Drosophila) | ATOH1 | -3.1 | NA | -3.2 |
| ENSG00000122859 | neurogenin 3 | NEUROG3 | NA | -2.8 | -3.5 |
| ENSG00000169507 | solute carrier family 38, member 11 | SLC38A11 | -4.2 | -2.9 | -2.4 |
| ENSG00000007952 | NADPH oxidase 1 | NOX1 | -4.2 | -2.9 | -2.1 |
| ENSG00000184564 | SLIT and NTRK-like family, member 6 | SLITRK6 | -3.0 | -3.4 | -1.8 |
| ENSG00000134193 | regenerating islet-derived family, member 4 | REG4 | -6.3 | -3.1 | -0.7 |
| ENSG00000104537 | annexin A13 | ANXA13 | -3.1 | -2.6 | -2.6 |
| ENSG00000188175 | HEPACAM family member 2 | HEPACAM2 | -2.2 | -2.7 | -3.0 |
| ENSG00000176024 | zinc finger protein 613 | ZNF613 | -1.7 | -3.3 | -2.3 |
| ENSG00000003989 | solute carrier family 7, member 2 | SLC7A2 | -3.4 | -1.7 | -2.6 |
| ENSG00000180745 | clarin 3 | CLRN3 | -3.3 | -2.8 | -1.7 |
| ENSG00000144485 | hes family bHLH transcription factor 6 | HES6 | -2.0 | -2.2 | -3.0 |
| ENSG00000101311 | fermitin family member 1 | FERMT1 | -2.4 | -2.6 | -2.1 |
| ENSG00000106003 | LFNG O-fucosylpeptide 3-beta-N-acetylglucosaminyltransferase | LFNG | -2.7 | -2.5 | -1.9 |
| ENSG00000198719 | delta-like 1 (Drosophila) | DLL1 | -2.2 | -2.7 | -2.0 |
| ENSG00000155792 | DEP domain containing MTOR-interacting protein | DEPTOR | -3.0 | -2.4 | -1.8 |
| ENSG00000232931 | long intergenic non-protein coding RNA 342 | LINC00342 | -0.9 | -3.1 | -2.9 |
| ENSG00000118513 | v-myb avian myeloblastosis viral oncogene homolog | MYB | -2.8 | -2.2 | -2.0 |
| ENSG00000173546 | chondroitin sulfate proteoglycan 4 | CSPG4 | -3.4 | -2.1 | -1.6 |
| ENSG00000114248 | leucine rich repeat containing 31 | LRRC31 | -2.6 | -1.9 | -2.2 |
| ENSG00000168874 | atonal homolog 8 (Drosophila) | ATOH8 | -2.0 | -2.7 | -2.0 |
| ENSG00000249267 | long intergenic non-protein coding RNA 939 | RP5-916L7.1 | -1.6 | -2.9 | -2.1 |
| ENSG00000121966 | chemokine (C-X-C motif) receptor 4 | CXCR4 | -2.9 | -1.9 | -2.0 |
| ENSG00000169218 | R-spondin 1 | RSPO1 | -2.3 | -4.0 | -0.4 |
| ENSG00000144579 | CTD (carboxy-terminal domain, RNA polymerase II, polypeptide A) small phosphatase 1 | CTDSP1 | -1.6 | -3.1 | -1.9 |
| ENSG00000215478 | carboxylesterase 5A pseudogene 1 | CES5AP1 | -4.0 | -1.9 | -1.2 |
| ENSG00000163501 | indian hedgehog | IHH | -3.4 | -3.2 | -0.5 |
| ENSG00000197408 | cytochrome P450, family 2, subfamily B, polypeptide 6 | CYP2B6 | -3.6 | -1.5 | -1.7 |
| ENSG00000265150 | RNA, 7SL, cytoplasmic 2 | RN7SL2 | -4.0 | -0.1 | -2.5 |
| ENSG00000157399 | arylsulfatase E (chondrodysplasia punctata 1) | ARSE | -2.9 | -2.2 | -1.6 |
| ENSG00000243766 | HOXA distal transcript antisense RNA | HOTTIP | -2.5 | -2.0 | -1.9 |
| ENSG00000157388 | calcium channel, voltage-dependent, L type, alpha 1D subunit | CACNA1D | -2.2 | -1.6 | -2.4 |
| ENSG00000214290 | colorectal cancer associated 2 | C11orf93 | -4.5 | -1.6 | -1.0 |
| ENSG00000196659 | tetratricopeptide repeat domain 30B | TTC30B | -1.2 | -2.9 | -2.1 |
| ENSG00000172086 | lysine-rich coiled-coil 1 | KRCC1 | -1.4 | -2.9 | -1.9 |
| ENSG00000124766 | SRY (sex determining region Y)-box 4 | SOX4 | -1.6 | -1.9 | -2.6 |
| ENSG00000165092 | aldehyde dehydrogenase 1 family, member A1 | ALDH1A1 | -4.2 | -1.6 | -0.9 |
| ENSG00000128000 | zinc finger protein 780B | ZNF780B | -1.3 | -2.4 | -2.4 |
| ENSG00000198835 | gap junction protein, gamma 2, 47kDa | GJC2 | -2.4 | -2.5 | -1.4 |
| ENSG00000174586 | zinc finger protein 497 | ZNF497 | -0.6 | -3.2 | -2.2 |
| ENSG00000198890 | protein arginine methyltransferase 6 | PRMT6 | -0.4 | -3.6 | -1.7 |
| ENSG00000186376 | zinc finger protein 75D | ZNF75D | -1.6 | -2.4 | -2.1 |

**Supplementary Table 4.** Top 50 most enriched gene ontology biological processes in non-irradiated versus 10x 2 Gy HT29 cells *in vitro*. GO = gene ontology, count = number of induced genes in the sample, size = total number of genes in the GO term.

| **GO** | **Term** | **Count** | **Size** |
| --- | --- | --- | --- |
| 0060337 | type I interferon-mediated signaling pathway | 19 | 75 |
| 0019221 | cytokine-mediated signaling pathway | 23 | 181 |
| 0009615 | response to virus | 18 | 144 |
| 0006955 | immune response | 27 | 382 |
| 0007267 | cell-cell signaling | 20 | 242 |
| 0007165 | signal transduction | 41 | 1176 |
| 0060333 | interferon-gamma-mediated signaling pathway | 11 | 82 |
| 0043066 | negative regulation of apoptotic process | 17 | 272 |
| 0008285 | negative regulation of cell proliferation | 19 | 341 |
| 0006334 | nucleosome assembly | 11 | 102 |
| 0006950 | response to stress | 13 | 155 |
| 0006916 | anti-apoptosis | 14 | 200 |
| 0008283 | cell proliferation | 17 | 312 |
| 0001666 | response to hypoxia | 13 | 175 |
| 0006986 | response to unfolded protein | 8 | 51 |
| 0007155 | cell adhesion | 23 | 556 |
| 0001525 | angiogenesis | 12 | 160 |
| 0030335 | positive regulation of cell migration | 10 | 105 |
| 0007568 | aging | 10 | 114 |
| 0006935 | chemotaxis | 10 | 126 |
| 0042493 | response to drug | 15 | 301 |
| 0045087 | innate immune response | 15 | 309 |
| 0019882 | antigen processing and presentation | 7 | 54 |
| 0006915 | apoptotic process | 21 | 594 |
| 0014070 | response to organic cyclic compound | 9 | 112 |
| 0051085 | chaperone mediated protein folding requiring cofactor | 4 | 11 |
| 0006952 | defense response | 7 | 67 |
| 0006508 | proteolysis | 19 | 543 |
| 0045672 | positive regulation of osteoclast differentiation | 4 | 14 |
| 0006914 | autophagy | 6 | 48 |
| 0007596 | blood coagulation | 17 | 457 |
| 0015992 | proton transport | 6 | 50 |
| 0032480 | negative regulation of type I interferon production | 5 | 30 |
| 0033572 | transferrin transport | 5 | 30 |
| 0044419 | interspecies interaction between organisms | 14 | 328 |
| 0032020 | ISG15-protein conjugation | 3 | 6 |
| 0042117 | monocyte activation | 3 | 6 |
| 0009308 | amine metabolic process | 3 | 6 |
| 0030199 | collagen fibril organization | 5 | 32 |
| 0015991 | ATP hydrolysis coupled proton transport | 5 | 32 |
| 0008633 | activation of pro-apoptotic gene products | 5 | 32 |
| 0045071 | negative regulation of viral genome replication | 4 | 16 |
| 0000079 | regulation of cyclin-dependent protein kinase activity | 6 | 55 |
| 0030308 | negative regulation of cell growth | 8 | 113 |
| 0010718 | positive regulation of epithelial to mesenchymal transition | 4 | 18 |
| 0060317 | cardiac epithelial to mesenchymal transition | 3 | 7 |
| 0032727 | positive regulation of interferon-alpha production | 3 | 7 |
| 0050714 | positive regulation of protein secretion | 4 | 19 |
| 0008219 | cell death | 9 | 156 |

**Supplementary Table 5.** Genes with an average log(2) fold change ≤ -1 or ≥ 1 (adjusted p-value < 0.10) as identified by human micro-array analysis of 9x 2 Gy vs. non-irradiated HT29 xenografts (n=4).

| **Gene ID** | **Name** | **Symbol** | **Log(2) fold change** |
| --- | --- | --- | --- |
| ILMN_1674063 | 2-5-oligoadenylate synthetase 2 | OAS2 | 3.73 |
| ILMN_1801246 | interferon induced transmembrane protein 1 | IFITM1 | 3.41 |
| ILMN_1803945 | HLA complex P5 | HCP5 | 3.15 |
| ILMN_1760062 | interferon-induced protein 44 | IFI44 | 3.05 |
| ILMN_2231928 | myxovirus (influenza virus) resistance 2 | MX2 | 2.78 |
| ILMN_1707695 | interferon-induced protein with tetratricopeptide repeats 1 | IFIT1 | 2.76 |
| ILMN_1662358 | myxovirus resistance 1, interferon-inducible protein p78 | MX1 | 2.56 |
| ILMN_1769520 | ubiquitin-conjugating enzyme E2L 6 | UBE2L6 | 2.54 |
| ILMN_1739428 | interferon-induced protein with tetratricopeptide repeats 2 | IFIT2 | 2.45 |
| ILMN_1778401 | major histocompatibility complex, class I, B | HLA-B | 2.30 |
| ILMN_2347798 | interferon, alpha-inducible protein 6 | IFI6 | 2.26 |
| ILMN_1795181 | DEAD (Asp-Glu-Ala-Asp) box polypeptide 60 | DDX60 | 2.16 |
| ILMN_1657871 | radical S-adenosyl methionine domain containing 2 | RSAD2 | 2.16 |
| ILMN_1687384 | interferon, alpha-inducible protein 6 | IFI6 | 1.96 |
| ILMN_2384857 | dehydrogenase/reductase (SDR family) member 2 | DHRS2 | 1.94 |
| ILMN_2058782 | interferon, alpha-inducible protein 27 | IFI27 | 1.89 |
| ILMN_2239754 | interferon-induced protein with tetratricopeptide repeats 3 | IFIT3 | 1.85 |
| ILMN_1703337 | hypothetical LOC441763 | LOC441763 | 1.75 |
| ILMN_1805750 | interferon induced transmembrane protein 3 (1-8U) | IFITM3 | 1.65 |
| ILMN_3239606 | hypothetical LOC100134357 | LOC100134357 | 1.54 |
| ILMN_1745397 | 2-5-oligoadenylate synthetase 3 | OAS3 | 1.54 |
| ILMN_1752622 | prospero homeobox 1 | PROX1 | 1.48 |
| ILMN_1751079 | transporter 1, ATP-binding cassette, sub-family B | TAP1 | 1.46 |
| ILMN_2262044 | poly (ADP-ribose) polymerase family, member 10 | PARP10 | 1.40 |
| ILMN_2053527 | poly (ADP-ribose) polymerase family, member 9 | PARP9 | 1.20 |
| ILMN_1755173 | pleckstrin homology domain containing, family A, member 4 | PLEKHA4 | 1.20 |
| ILMN_2305112 | cystathionase (cystathionine gamma-lyase) | CTH | 1.14 |
| ILMN_1710303 | tetratricopeptide repeat domain 25 | TTC25 | 1.03 |
| ILMN_2108735 | eukaryotic translation elongation factor 1 alpha 2 | EEF1A2 | 1.02 |
| ILMN_1762769 | polymerase (RNA) II (DNA directed) polypeptide J | POLR2J4 | -1.09 |
| ILMN_3200018 | similar to hCG1815881 | LOC442609 | -1.12 |
| ILMN_1655864 | similar to hypothetical protein FLJ40722, transcript variant 2 | LOC653853 | -1.38 |
| ILMN_1742917 | nucleoredoxin-like 1 | NXNL1 | -1.69 |
| ILMN_1667796 | hemoglobin, alpha 2 | HBA2 | -2.68 |

**Supplementary Table 6.** Top 50 most enriched gene ontology biological processes in non-irradiated versus 9x 2 Gy HT29 xenografts. GO = gene ontology, count = number of induced genes in the sample, size = total number of genes in the GO term.

| **GO** | **Term** | **Count** | **Size** |
| --- | --- | --- | --- |
| 0060337 | type I interferon-mediated signaling pathway | 18 | 61 |
| 0071357 | cellular response to type I interferon | 18 | 61 |
| 0034340 | response to type I interferon | 18 | 62 |
| 0051607 | defense response to virus | 22 | 149 |
| 0045087 | innate immune response | 32 | 383 |
| 0045071 | negative regulation of viral genome replication | 11 | 30 |
| 0048525 | negative regulation of viral reproduction | 11 | 30 |
| 0009615 | response to virus | 23 | 209 |
| 0006955 | immune response | 42 | 722 |
| 0043901 | negative regulation of multi-organism process | 11 | 40 |
| 0045069 | regulation of viral genome replication | 11 | 44 |
| 0002252 | immune effector process | 25 | 318 |
| 0019079 | viral genome replication | 11 | 53 |
| 0009607 | response to biotic stimulus | 28 | 419 |
| 0019221 | cytokine-mediated signaling pathway | 22 | 265 |
| 0006952 | defense response | 39 | 752 |
| 2000242 | negative regulation of reproductive process | 11 | 56 |
| 0051707 | response to other organism | 27 | 402 |
| 0002376 | immune system process | 54 | 1321 |
| 0071345 | cellular response to cytokine stimulus | 23 | 323 |
| 0035455 | response to interferon-alpha | 6 | 14 |
| 0043900 | regulation of multi-organism process | 16 | 176 |
| 0034097 | response to cytokine stimulus | 24 | 391 |
| 0039531 | regulation of viral-induced cytoplasmic pattern recognition receptor signaling pathway | 4 | 6 |
| 0039535 | regulation of RIG-I signaling pathway | 4 | 6 |
| 0035457 | cellular response to interferon-alpha | 4 | 7 |
| 0039529 | RIG-I signaling pathway | 4 | 7 |
| 0050792 | regulation of viral reproduction | 11 | 105 |
| 0034341 | response to interferon-gamma | 10 | 86 |
| 1900246 | positive regulation of RIG-I signaling pathway | 3 | 3 |
| 0006950 | response to stress | 69 | 2249 |
| 0039528 | cytoplasmic pattern recognition receptor signaling pathway in response to virus | 4 | 10 |
| 0035456 | response to interferon-beta | 4 | 13 |
| 0050688 | regulation of defense response to virus | 7 | 56 |
| 0070887 | cellular response to chemical stimulus | 44 | 1310 |
| 0032020 | ISG15-protein conjugation | 3 | 6 |
| 2000241 | regulation of reproductive process | 11 | 157 |
| 0071310 | cellular response to organic substance | 36 | 1031 |
| 0001817 | regulation of cytokine production | 15 | 277 |
| 0002831 | regulation of response to biotic stimulus | 7 | 69 |
| 0010212 | response to ionizing radiation | 8 | 91 |
| 0002682 | regulation of immune system process | 25 | 629 |
| 0060700 | regulation of ribonuclease activity | 2 | 2 |
| 1900245 | positive regulation of MDA-5 signaling pathway | 2 | 2 |
| 0031347 | regulation of defense response | 16 | 322 |
| 0043331 | response to dsRNA | 5 | 34 |
| 0071346 | cellular response to interferon-gamma | 7 | 73 |
| 0045088 | regulation of innate immune response | 11 | 178 |
| 2000116 | regulation of cysteine-type endopeptidase activity | 10 | 153 |

**Supplementary Table 7.** Baseline characteristics esophageal cancer patients.

|  | **Cohort 1** | **Cohort 2** | **Cohort 3** | **Cohort 4** | **Total** |  |
| --- | --- | --- | --- | --- | --- | --- |
| **Age*** | 64 (57-75) | 68 (54-71) | 69 (46-71) | 70 (65-75) | 68.5 (46-75) |  |
| **Gender** |  |  |  |  |  |  |
| *male* | 5 | 5 | 3 | 5 | 18 | 75.0% |
| *female* | 0 | 1 | 2 | 0 | 3 | 12.5% |
| *Missing* | 2 | 0 | 1 | 0 | 3 | 12.5% |
| **Histology** |  |  |  |  |  |  |
| *EAC^#^* | 7 | 5 | 5 | 4 | 21 | 87.5% |
| *ESCC^##^* | 0 | 1 | 0 | 1 | 2 | 8.3% |
| *Missing* | 0 | 0 | 1 | 0 | 1 | 4.2% |
| **Fractions of RTx** | 5 (3-7) | 11.5 (10-12) | 15.5 (14-17) | 22 (20-22) | 12 (3-22) |  |
| **T stage** |  |  |  |  |  |  |
| *T1* | 0 | 0 | 0 | 0 | 0 | 0.0% |
| *T2* | 1 | 1 | 0 | 0 | 2 | 8.3% |
| *T3* | 6 | 5 | 5 | 5 | 21 | 87.5% |
| *Missing* | 0 | 0 | 1 | 0 | 1 | 4.2% |
| **N stage** |  |  |  |  |  |  |
| *N0* | 2 | 3 | 1 | 3 | 9 | 37.5% |
| *N1* | 4 | 1 | 2 | 1 | 8 | 33.3% |
| *N2* | 1 | 1 | 2 | 0 | 4 | 16.7% |
| *N3* | 0 | 0 | 0 | 1 | 1 | 4.2% |
| *Missing* | 0 | 0 | 1 | 0 | 2 | 8.3% |
| **Total** | 7 | 6 | 6 | 5 | 24 | 100.0% |
| **Excluded**** | 1 | 1 | 2 | 0 | 4 | 17.7% |

* Median + range is shown for age and fractions of radiotherapy (RTx)

# Esophageal adenocarcinoma

## Esophageal squamous cell carcinoma

** Excluded after assessment of representative tumor biopsy area on H&E stain (NvG).
