# Supplementary material for: Interferon- and STING-independent induction of type I interferon stimulated genes during fractionated irradiation
Source: J Exp Clin Cancer Res. 2021 May 8;40:161. doi: 10.1186/s13046-021-01962-2 (PMC8106844; doi:10.1186/s13046-021-01962-2)
Supplement: Supplementary file 2 — Additional file 2. [file 13046_2021_1962_MOESM2_ESM.zip › Additional file 2 Supplementary figure S1 to S8 legends.docx]

**Supplementary figure legends**

**Supplementary Figure S1**

Dotplot showing the top 30 most significantly upregulated Gene Ontology (GO) terms (biological processes) after 2 weeks of treatment vs. untreated based on RNA deep-sequencing of HT29 cells.

Same as (a), but with downregulated GO terms. * regulation of transcription in G1/S phase of mitotic cell cycle.

REVIGO treemap summarizing upregulated and related non-redundant GO terms (biological processes) after 2 weeks of treatment vs. untreated based on RNA deep-sequencing of HT29 cells.

**Supplementary Figure S2**

Bar graphs showing fold change in mRNA expression levels of a panel of ISGs in different cancer cell lines subjected to 10x 2 Gy as compared to no irradiation. SW480, HCT116, COLO320 and RKO are colon adenocarcinoma cell lines, D384 is a glioma cell line. * p-value ≤ 0.05 vs. no irradiation.

**Supplementary Figure S3**

Time course analysis of ISG mRNA expression in D384 glioma cells. Data are expressed as fold-change vs. 0x 2 Gy irradiation (n=2).

Bar graphs showing fold change in mRNA expression levels of a panel of ISGs in HT29 cells subjected to single dose irradiation with increasing dose fraction.

**Supplementary Figure S4**

Tumor volume (mm^3^) of HT29 xenograft tumors with (black squares) or without (white squares) irradiation after 0, 2, 4, 6, 8, 12 and 14 fractions of 2 Gy, compared to 10 fractions (day 14, dashed line). Note that the volumes of irradiated tumors are not significantly different after 8-14 fractions, whereas the volume of non-irradiated tumors has significantly increased after 18 days compared to day 14. * p-value ≤ 0.05 vs. day 14/10x 2Gy

Dotplot showing the top 30 most significantly upregulated Gene Ontology (GO) terms (biological processes) in HT29 xenograft tumors after 2 weeks of RTx vs. no irradiation based on human microarray analysis.

Volcano plots of microarray data comparing gene expression in HT29 xenograft tumors after 1, 2, 3 weeks and a single dose (SD) of 5 Gy of irradiation vs. no irradiation. NS = not significant. FC = fold change.

Bar graphs showing fold change in mRNA expression levels of a panel of ISGs in HT29 xenograft tumor subjected to 9x 2 Gy irradiation vs. non-irradiated tumors. n=5 mice/group. * p-value ≤ 0.05 vs. non-irradiated tumors.

**Supplementary Figure S5**

Bar graphs showing fold change in mRNA expression levels of different IFNs in multiple colon adenocarcinoma cancer cell lines during fractionated irradiation. Fold difference vs. non-irradiated cells is shown. For comparison, the scaling of the y-axis is set to a similar range for each specific IFN (columns).

Bar graphs showing detectable expression of IFNAR1/2 (type I IFN receptors) as well as of IFNLR and IL10bR (type III IFN receptors) in all cell lines.

**Supplementary Figure S6**

mRNA expression levels of ISGs in patient-matched tumor samples from esophageal cancer patients (n=20) prior to or during chemoradiotherapy. The levels in the different cohorts are shown, i.e. 1 week (n= 6), 2 weeks (n= 5), 3 weeks (n= 4), 4 weeks (n= 5). * p-value ≤ 0.05 vs. matched pre-treatment samples. Fold expression in on-treatment samples vs. pre-treatment is shown.

Correlation plot showing the significant correlations between IFN and ISG induction levels from esophageal cancer patients during chemoradiotherapy (Pearson correlation coefficient only shown if p-value ≤ 0.05).

**Supplementary Figure S7**

Western blot of cGAS and STING in RKO cells (which lack STING) and HCT116 cells (which lack cGAS) subject to single dose irradiation. Irradiation did not induce expression of the absent proteins. THP-1 represents the positive control for cGAS and STING, Actin staining was used as loading control.

Dose response analyses of effect of anti IFN-β (left panel) and anti IFN-λ (right panel) on growth (MTT assay) of different colon cell lines.

Western blot of STING and phosphorylated TBK1 in cells subject to 0 Gy or 4 Gy irradiation in the absence or presence of increasing concentrations of the STING antagonist H151. When no irradiation is applied (upper panel) TBK1 phosphorylation is low and is not affected by H151. When cells are irradiated (lower panel) TBK1 phosphorylation is increased in the absence of H151 but inhibited in the presence of H151. This indicates effective STING inhibition by H151.

Effect of either anti IFN-β (white dots), anti IFN-λ (light grey dots) or H151 (dark grey dots) on basal clonogenic survival of HT29 cells. Neither treatment significantly affects clonogenic survival in non-irradiated cells.

Effect of either anti IFN-β, anti IFN-λ or H151 on HT29 cell numbers during fractionated irradiation. Starting from 10x 2 Gy, anti IFN-β antibody prevented inhibition of cell growth, in line with the known growth inhibitory role of IFN-β and the observed induction of IFN-β from 10x 2 Gy onwards. No effect of anti IFN-λ treatment or STING antagonist H151 could be observed.

**Supplementary Figure S8**

Western blot of STING on HT29 wild type cells and 10 different knockout clones.

Schematic representation of the STING gene. Exons are shown as boxes and introns as lines. Grey areas represent protein coding regions and white areas represent untranslated regions. The dotted line indicates the target region of the CRISPR/CAS guide. Below, the sequence of the wild-type HT29 cells and one of the HT29 STING knockout cells is shown. CRISPR/CAS gene editing resulted in insertion of a thymidine causing a frame shift resulting in two premature and adjacent STOP codons (shown as asterisks).
