# Supplementary figures and images for: Interferon- and STING-independent induction of type I interferon stimulated genes during fractionated irradiation
Source: J Exp Clin Cancer Res. 2021 May 8;40:161. doi: 10.1186/s13046-021-01962-2 (PMC8106844; doi:10.1186/s13046-021-01962-2)

Supplementary figure S1

a Top 30 upregulated GO terms

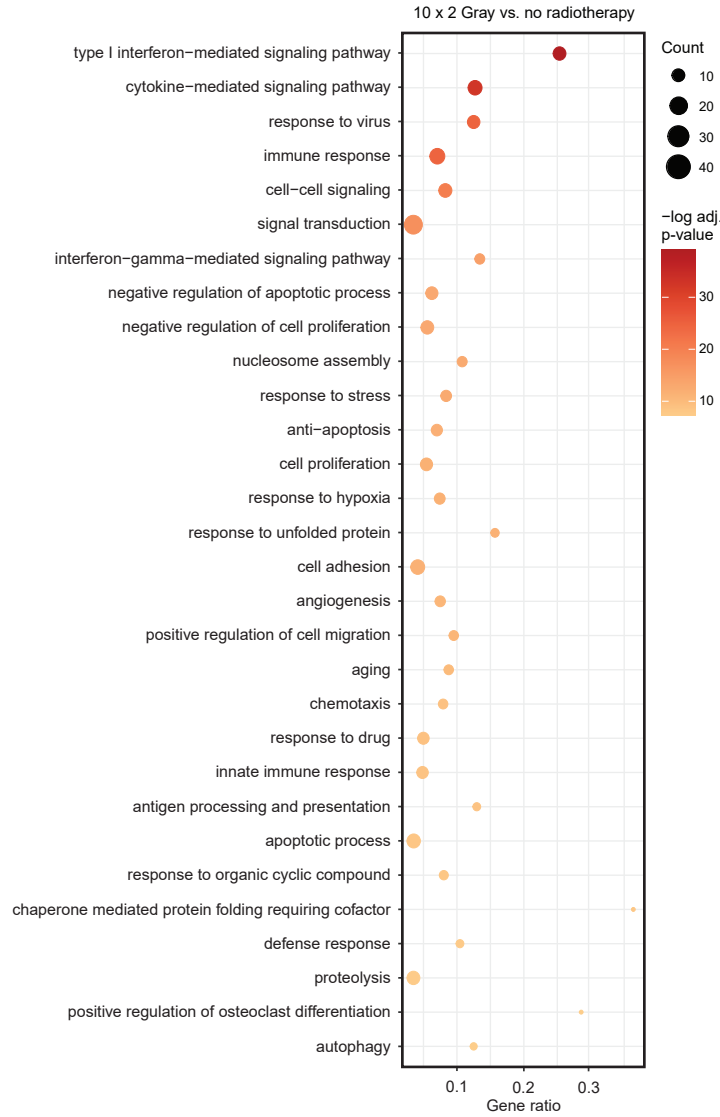

b Top 30 downregulated GO terms

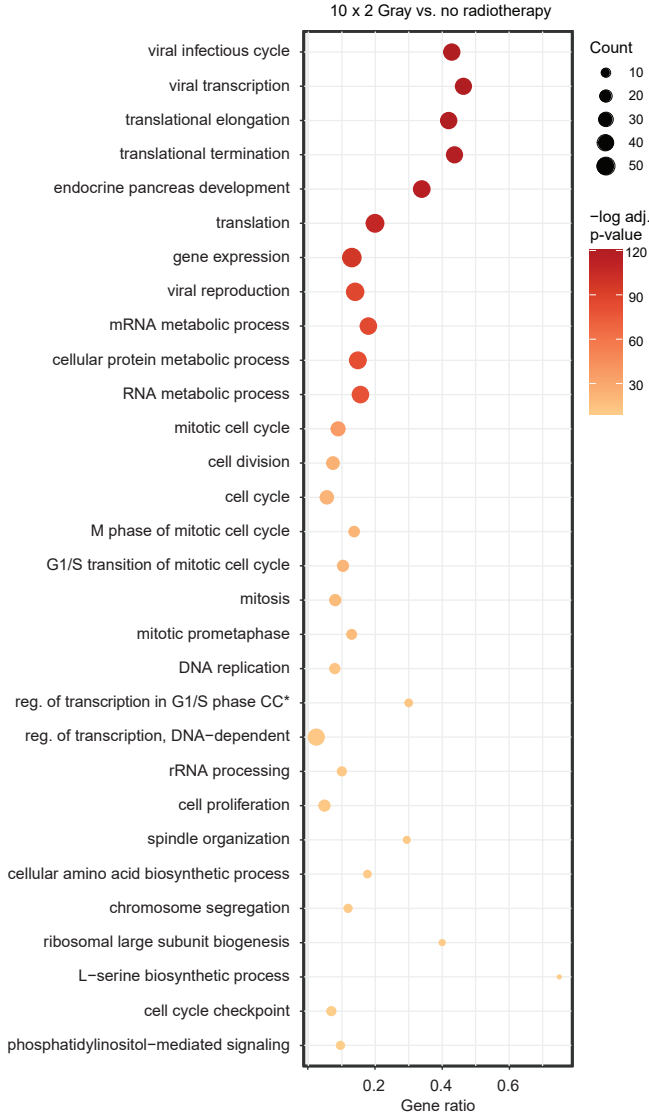

Supplement: Supplementary file 2 — Additional file 2. [file 13046_2021_1962_MOESM2_ESM.zip › Figure S1.pdf]

Supplementary figure S2

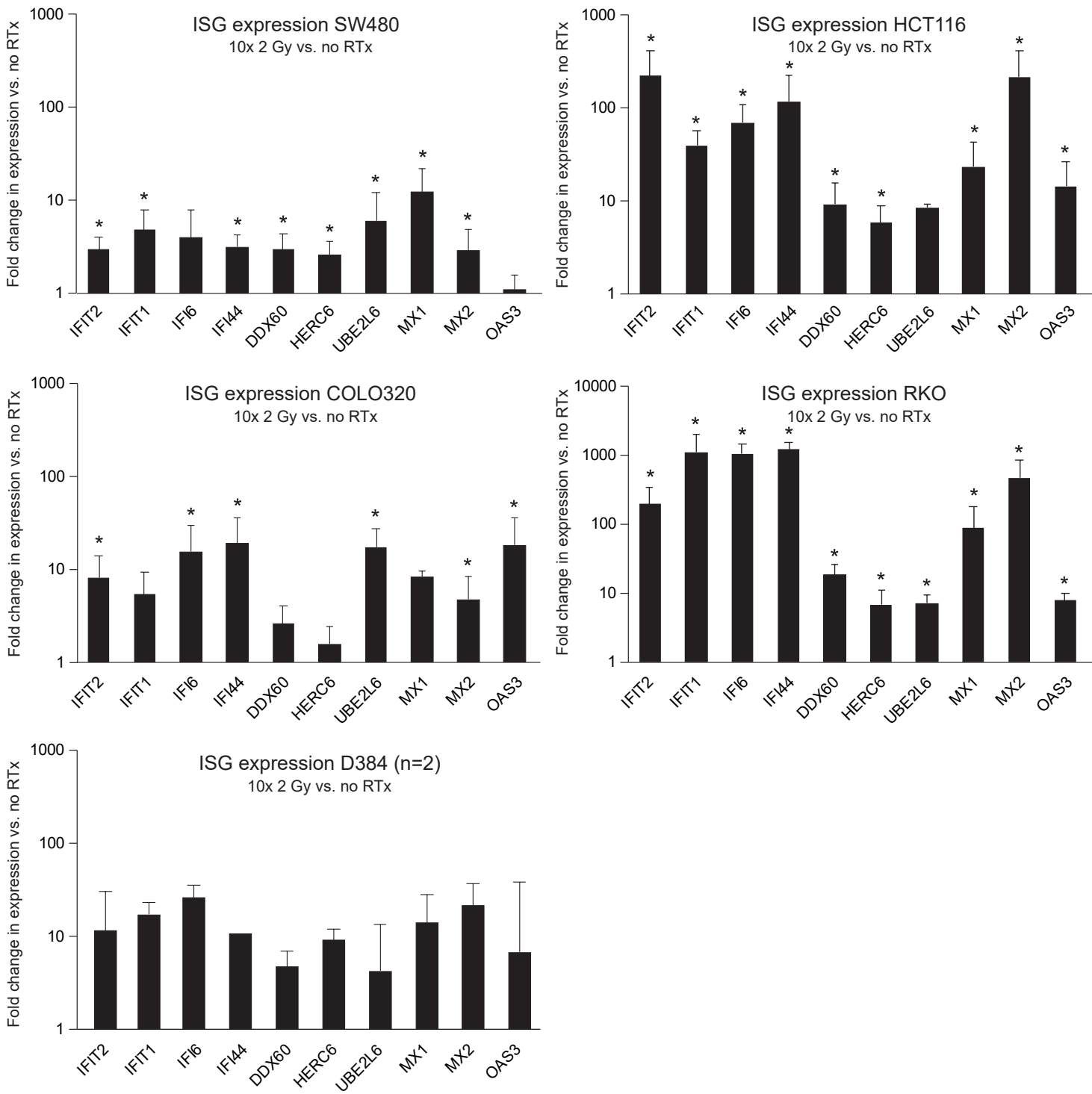

Supplement: Supplementary file 2 — Additional file 2. [file 13046_2021_1962_MOESM2_ESM.zip › Figure S2.pdf]

Supplementary figure S3

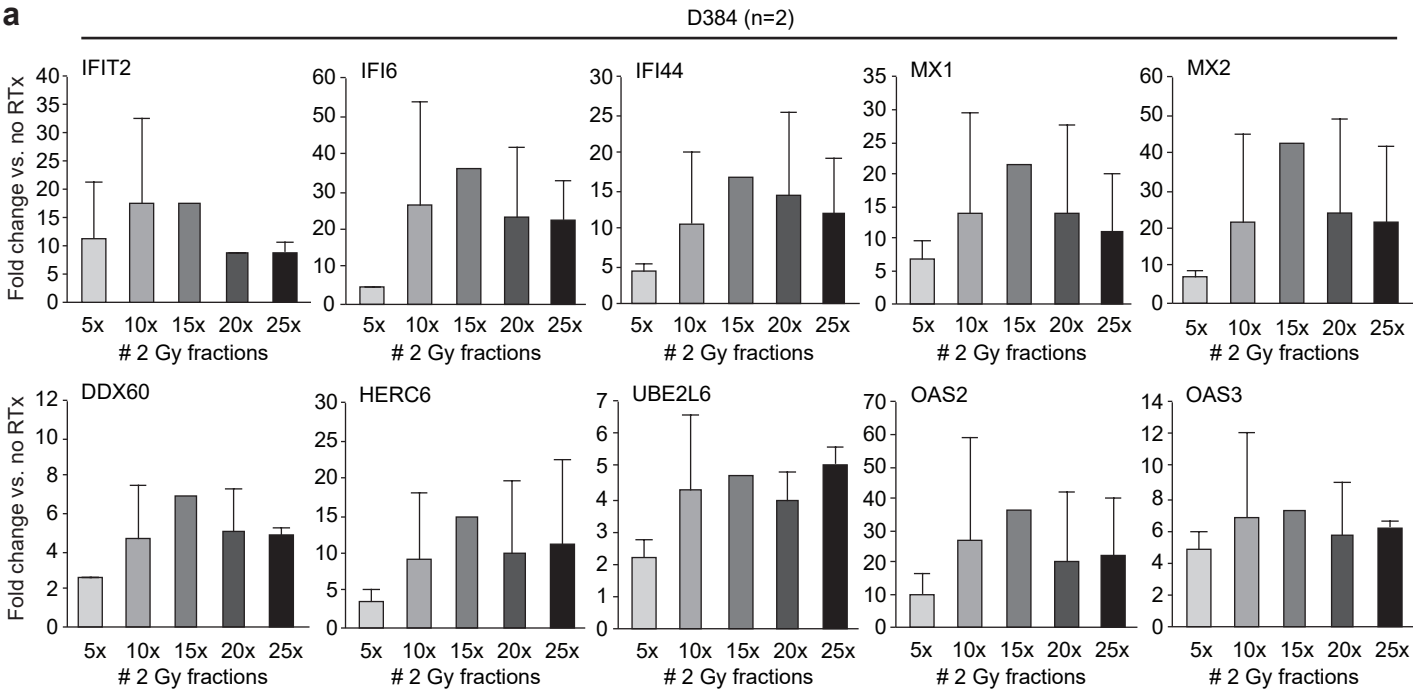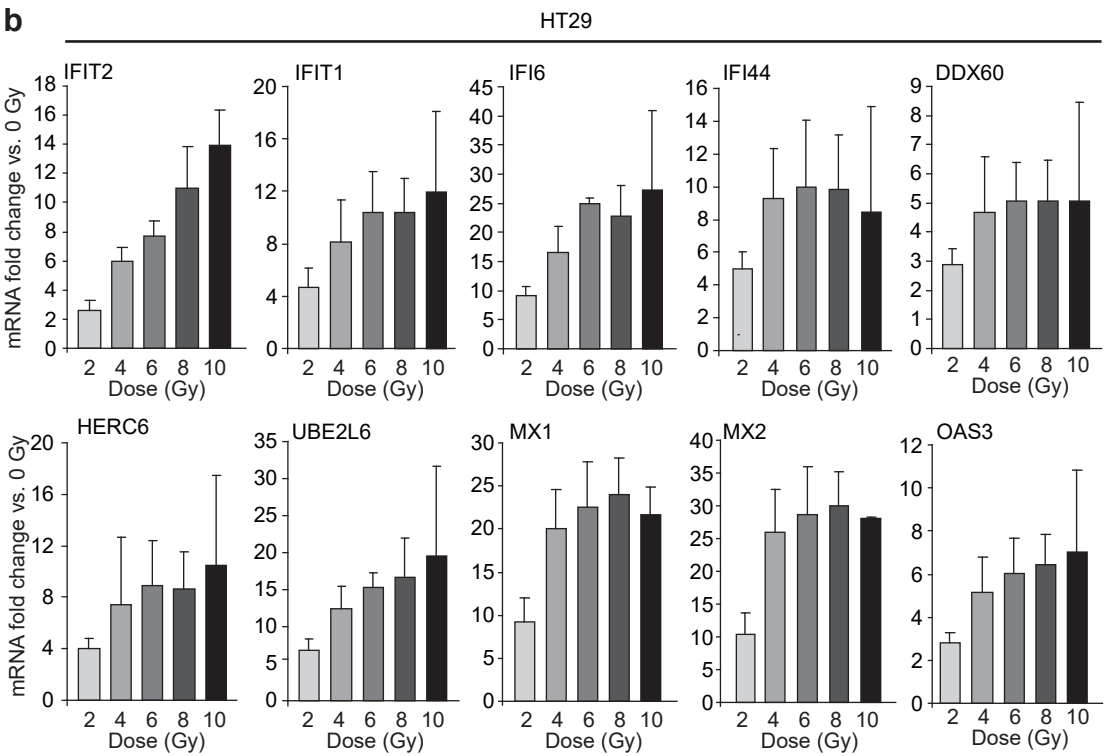

Supplement: Supplementary file 2 — Additional file 2. [file 13046_2021_1962_MOESM2_ESM.zip › Figure S3.pdf]

Supplementary figure S4

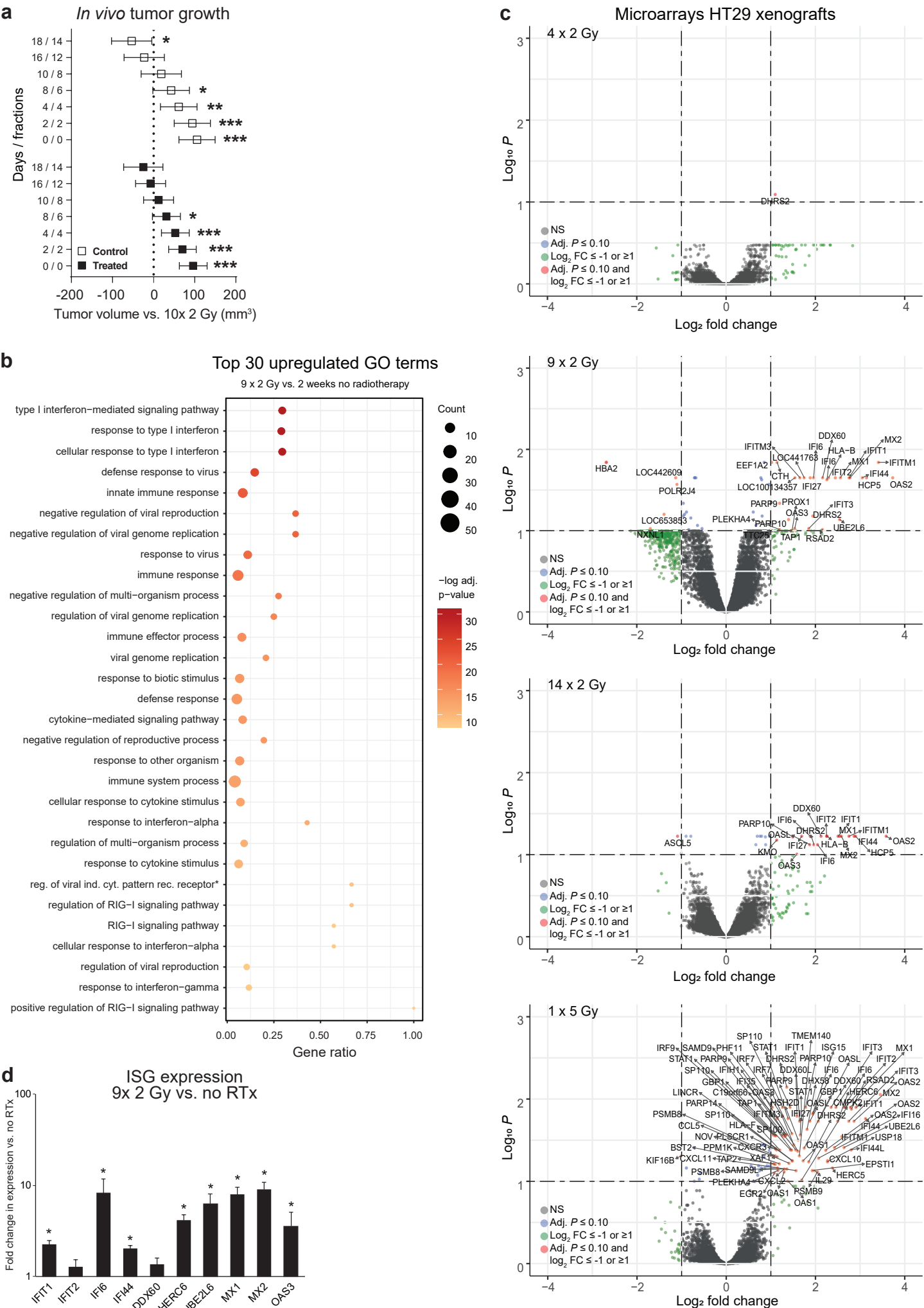

Supplement: Supplementary file 2 — Additional file 2. [file 13046_2021_1962_MOESM2_ESM.zip › Figure S4.pdf]

Supplementary figure S5

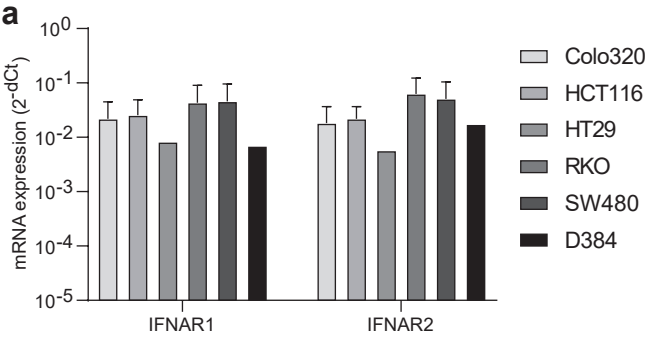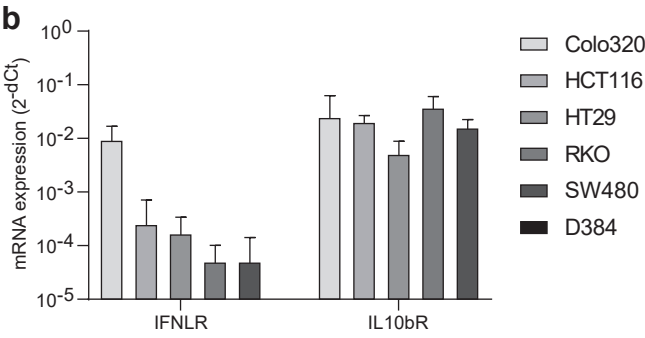

Supplement: Supplementary file 2 — Additional file 2. [file 13046_2021_1962_MOESM2_ESM.zip › Figure S5.pdf]

### Supplementary figure S6

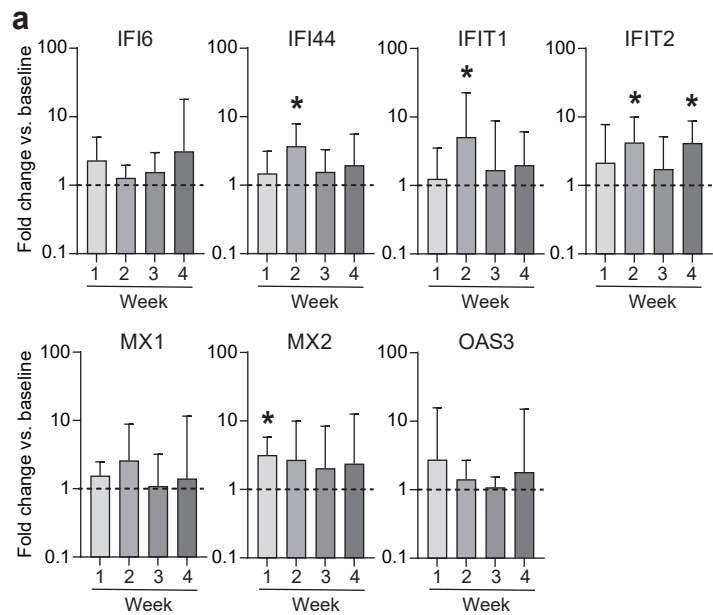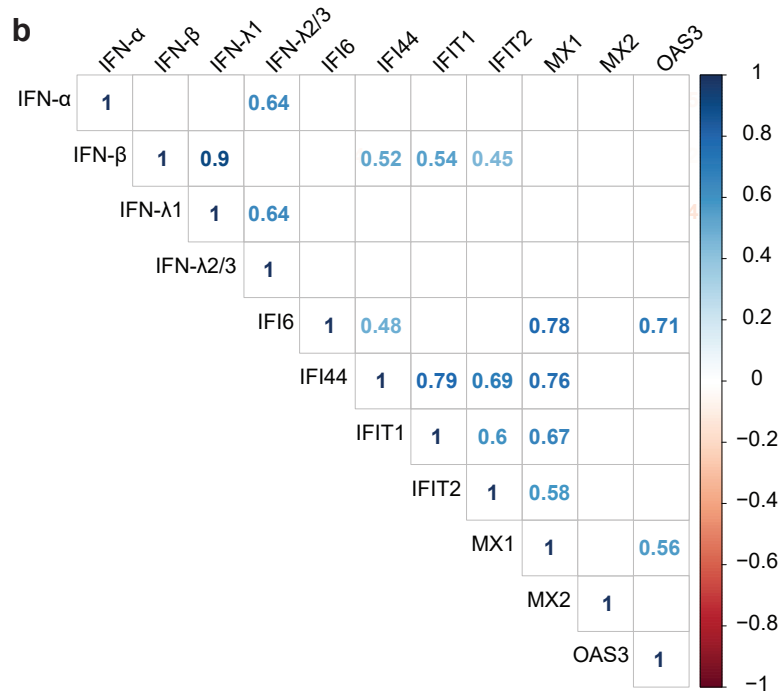

Supplement: Supplementary file 2 — Additional file 2. [file 13046_2021_1962_MOESM2_ESM.zip › Figure S6.pdf]

Supplementary figure S7

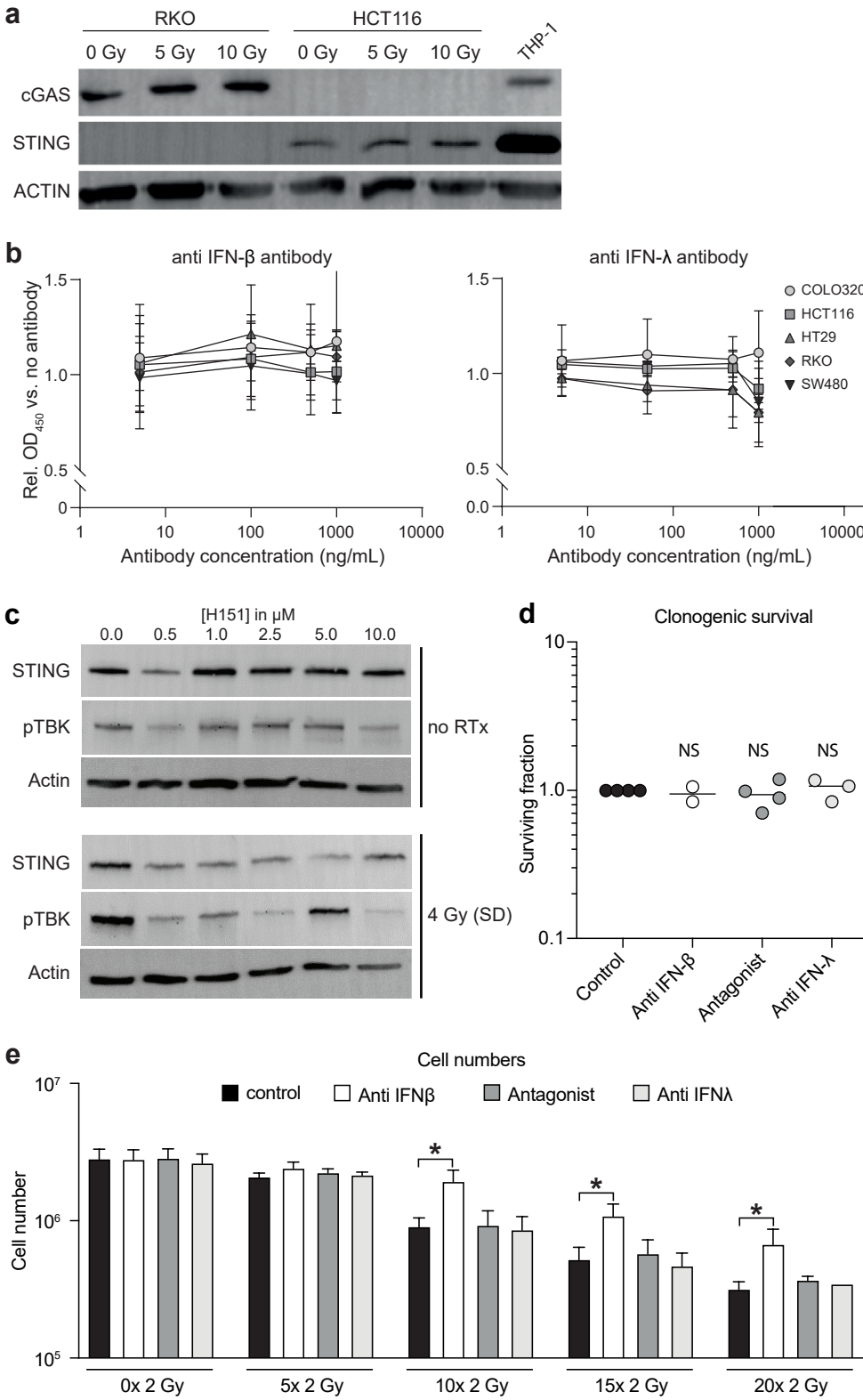

Supplement: Supplementary file 2 — Additional file 2. [file 13046_2021_1962_MOESM2_ESM.zip › Figure S7.pdf]

Supplementary figure S8

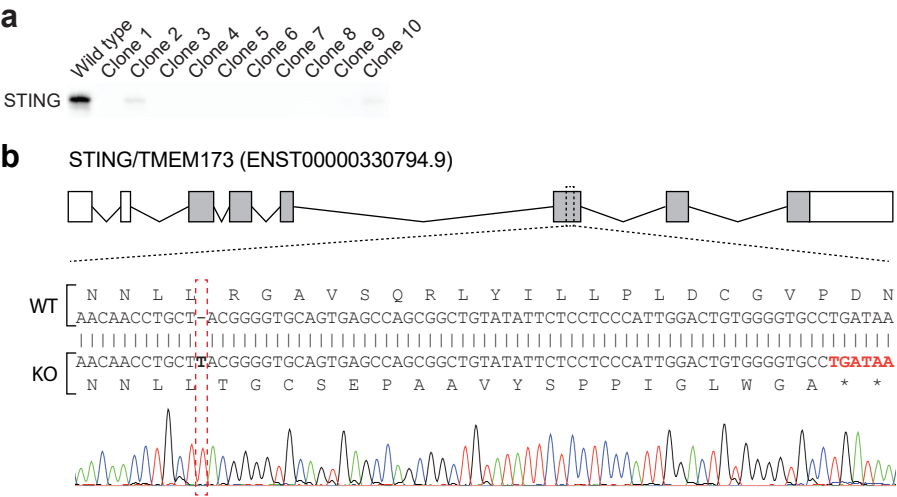

Supplement: Supplementary file 2 — Additional file 2. [file 13046_2021_1962_MOESM2_ESM.zip › Figure S8.pdf]
